# Spatio-Temporal Detection of the *Thiomonas* Population and the *Thiomonas* Arsenite Oxidase Involved in Natural Arsenite Attenuation Processes in the Carnoulès Acid Mine Drainage

**DOI:** 10.3389/fcell.2016.00003

**Published:** 2016-02-01

**Authors:** Agnès Hovasse, Odile Bruneel, Corinne Casiot, Angélique Desoeuvre, Julien Farasin, Marina Hery, Alain Van Dorsselaer, Christine Carapito, Florence Arsène-Ploetze

**Affiliations:** ^1^Laboratoire de Spectrométrie de Masse BioOrganique, Institut Pluridisciplinaire Hubert Curien, UMR7178, Centre National de la Recherche Scientifique, Université de StrasbourgStrasbourg, France; ^2^Laboratoire HydroSciences Montpellier, UMR HSM 5569, Centre National de la Recherche Scientifique, Institut de Recherche pour le Développement, Université MontpellierMontpellier, France; ^3^Laboratoire Génétique Moléculaire, Génomique et Microbiologie, UMR7156, Centre National de la Recherche Scientifique-Université de Strasbourg, Département Microorganismes, Génomes, Environnement, Equipe Ecophysiologie Moléculaire des MicroorganismesStrasbourg, France

**Keywords:** community proteomics, targeted proteomics, LC-SRM, acid mine drainage, pyrosequencing, FISH

## Abstract

The acid mine drainage (AMD) impacted creek of the Carnoulès mine (Southern France) is characterized by acid waters with a high heavy metal content. The microbial community inhabiting this AMD was extensively studied using isolation, metagenomic and metaproteomic methods, and the results showed that a natural arsenic (and iron) attenuation process involving the arsenite oxidase activity of several *Thiomonas* strains occurs at this site. A sensitive quantitative Selected Reaction Monitoring (SRM)-based proteomic approach was developed for detecting and quantifying the two subunits of the arsenite oxidase and RpoA of two different *Thiomonas* groups. Using this approach combined with FISH and pyrosequencing-based 16S rRNA gene sequence analysis, it was established here for the first time that these *Thiomonas* strains are ubiquitously present in minor proportions in this AMD and that they express the key enzymes involved in natural remediation processes at various locations and time points. In addition to these findings, this study also confirms that targeted proteomics applied at the community level can be used to detect weakly abundant proteins *in situ*.

## Introduction

Arsenic contamination has been identified as a major risk to human health in various parts of the world (Mandal and Suzuki, 2002; Sharma and Sohn, 2009): more than 50 million people are exposed to high concentrations worldwide (Oremland and Stolz, 2003; Slyemi and Bonnefoy, 2012; Kruger et al., 2013). Acid mine drainage (AMD) environments are known to be highly toxic to most living organisms due to the presence of many toxic elements such as arsenic and because of the acidic pH, which is usually below 3. The sulfurous wastes of the former mine in Carnoulès, Gard (France) contain As-rich pyrite, which is continuously leached by subsurface waters emerging at the base of a dam from which the small Reigous Creek originates. This creek contains between 50 and 350 mg/L of soluble arsenic, mainly in the form of As(III) (Casiot et al., 2003; Morin et al., 2003; Egal et al., 2010). The arsenic concentration decreases by 95% between the source of the Reigous and its confluence with the downstream river Amous.

Despite these harsh conditions, a microbial community composed of bacteria, archaea, and eukaryotes has been known to exist for several years in this AMD and has been described in several studies (Bruneel et al., 2006, 2008, 2011; Volant et al., 2012, 2014). Microorganisms isolated from this site have been analyzed under laboratory conditions in order to determine their metabolic role in toxic ecosystems (Bruneel et al., 2003; Casiot et al., 2003, 2004; Duquesne et al., 2003, 2008; Morin et al., 2003; Bryan et al., 2009; Halter et al., 2012). In addition, seven bacterial genomes have been reconstructed using environmental genomic approaches (Bertin et al., 2011). These approaches combined with geochemical studies (Casiot et al., 2003; Morin et al., 2003; Egal et al., 2010) have shown that the natural attenuation process observed in the Reigous creek was mainly due to microbial metabolism. In particular, it has been suggested that *Acidithiobacillus* and *Gallionella* genera may be involved in the oxidation of iron into Fe(III), whereas *Thiomonas* strains oxidize arsenite (Casiot et al., 2003, 2005; Duquesne et al., 2003; Morin et al., 2003; Bruneel et al., 2006; Battaglia-Brunet et al., 2011a; Bertin et al., 2011).

Bacterial As(III) oxidation is of particular relevance to natural remediation processes since As(III) is more soluble and more toxic than As(V) (Oremland and Stolz, 2003; Kruger et al., 2013). The arsenite oxidase involved in these processes (AioA/B) is a dimethyl sulfoxide (DMSO) reductase, a member of the molybdenum family (Oremland and Stolz, 2003; Silver and Phung, 2005). These enzymes are heterodimers consisting of a large subunit with a molybdenum center and a [3Fe-4S] cluster associated with a small subunit with a Rieske-type [2Fe-2S] cluster. *aioA* and *aioB* genes have been found to exist in several phylogenetically diverse bacteria distributed among 25 genera, which have been isolated from various arsenic-rich environments (Quéméneur et al., 2008, 2010; Heinrich-Salmeron et al., 2011; Slyemi and Bonnefoy, 2012).

Eight strains, *Thiomonas* sp. X19, CB1, CB2, CB3, CB6, ACO3, ACO7, and *Thiomonas arsenitoxydans* (strain 3As), were isolated from the acidic drainage waters present in tailings of the former Carnoulès mine (Duquesne et al., 2008; Bryan et al., 2009; Delavat et al., 2012; Freel et al., 2015). *Thiomonas arsenivorans* was also isolated from another arsenic-rich mine residue at the former Cheni gold mine in the Limousin region, France (Battaglia-Brunet et al., 2006). The genomes of seven of these *Thiomonas* strains have been recently characterized (Freel et al., 2015). Phylogenetically, this genus is a member of the *Betaproteobacteria* subclass. It was proposed to attribute *Thiomonas* sp. CB1, CB2, CB3, CB6, ACO3, ACO7 along with *Tm. arsenitoxydans* to the same Group I clade, while *Thiomonas* sp. X19 belong to the Group II clade, along with *Tm. arsenivorans* isolated from the former Cheni gold mine (Bryan et al., 2009; Bertin et al., 2011; Freel et al., 2015). These *Thiomonas* strains show some interesting physiological and phylogenetic differences, and their ability to adapt to arsenic was also found to differ although some of these strains were isolated from the same environment (Battaglia-Brunet et al., 2006, 2011b; Bryan et al., 2009; Arsène-Ploetze et al., 2010; Freel et al., 2015). Interestingly, in several genomes of the *Thiomonas* strains isolated from the Reigous creek, at least two *aio* operons were detected on two genomic islands. Some of these *Thiomonas* strains were able to express at least two copies under laboratory conditions, and were highly resistant to arsenic (Freel et al., 2015). The expression of these genes has been found to occur only in the presence of arsenic (Duquesne et al., 2008; Slyemi et al., 2013; Freel et al., 2015).

Metagenomic data obtained on the Carnoulès AMD sediments made it possible to characterize the reconstructed genome of an additional bacterium (Carn2) belonging to the *Thiomonas* Group II (Bertin et al., 2011). In addition, environmental proteomic studies performed in May 2007 at one sampling point in the Reigous creek (COWG) showed that at least one *Thiomonas* strain belonging to Group II (Carn2) expressed the arsenite oxidase (Bertin et al., 2011). However, it has not been established so far whether these various *Thiomonas* strains are stably present at different places in the Reigous creek and whether they express the arsenite oxidase enzyme sustainably. In this study, environmental proteomic and genomic approaches were therefore developed and used to determine which of the *Thiomonas* strains are present and express arsenite oxidase at various locations and time points.

## Materials and methods

### Bacterial strains, growth conditions, and cell lysis

*Thiomonas* sp. CB2, CB1, CB3, and CB6 were isolated from the Reigous creek at the former Carnoulès mine (France) (Duquesne et al., 2008; Bryan et al., 2009; Arsène-Ploetze et al., 2010). *Thiomonas* strains were routinely grown on solid or liquid m126 medium (modified 126 medium: m126). Medium m126 containing (g/L) yeast extract (YE; 0.5); Na_2_S_2_O_3_ (5.0); KH_2_PO_4_ (1.5); Na_2_HPO_4_ (4.5); MgSO_4_·7H_2_O (0.1); and (NH_4_)Cl (0.3) was adjusted to pH 5.0 with H_2_SO_4_ before being sterilized. Arsenite (As(III)) from sterile stocks of 667.4 mM of the metalloid ion obtained from NaAsO_2_ (Prolabo) was added to this medium to obtain the concentration required. Cells grown from OD_600nm_ 0.002–0.2 (100 mL) were harvested by performing a 10-min centrifugation step (at 7000 g, at a temperature of 4°C). Pellets were washed once with NaCl (9 g/L) and suspended in 600 μL of ultra-pure water supplemented with 2 μL of Benzonase Nuclease (Sigma) and 4 μL of Protease Inhibitor Mix (GE Healthcare). Cell suspensions were then disrupted by adding glass beads (1 g) and vortexing them 3 times for 1 min with 30-s intervals. Cellular debris were removed by performing two centrifugation runs, at 10,000 g for 5 min and then at 16,000 g for 90 min. Protein concentrations were measured using the Qubit® Fluorometric Quantitation kit, in line with the manufacturer's instructions (Life technologies). The biological samples used in these experiments were prepared in triplicate.

### Sampling procedure

Samples were collected in June 2011 and January 2012 in several places along the Reigous creek: at the spring (S1), 30 m downstream of the spring (COWG), and 1500 m downstream of the spring (CONF), a few meters upstream of the confluence between the Reigous creek and the Amous river (Bruneel et al., 2003). Sediment samples (surface layer depth: 5 cm) were collected in triplicate from the bottom of the creek using a sterile spatula. The triplicate samples were pooled, homogenized, and placed on ice on the field. They were kept at 4°C for 7 days or frozen in the presence of 1 volume of 50% glycerol solution for analysis.

The main physicochemical parameters of the running water above the sediments (pH, temperature, and dissolved oxygen concentrations) were measured *in situ* at each sampling point (Table 1). Arsenic speciation, Fe(II) and sulfate analyses were performed as previously described (Bruneel et al., 2008; Egal et al., 2010). Briefly, water samples (500 mL) were immediately filtered through 0.22-μm Millipore membranes fitted to Sartorius polycarbonate filter holders. Prior to the total Fe and As determinations, the filtered water was acidified to pH 1 with HNO_3_ (14.5 M) and stored at 4°C in polyethylene bottles for analysis. A 20-μL aliquot of filtered water sample was added to either a mixture of acetic acid and EDTA for As speciation (Samanta and Clifford, 2005) or a mixture of 0.5 mL acetate buffer (pH 4.5) and 1 mL of 1,10-phenanthrolinium chloride solution for Fe(II) determination (Rodier et al., 1996). Deionized water was added to the vials to obtain a final volume of 10 ml. The samples to be used for Fe and As speciation and sulfate determination were stored in the dark and analyzed within 24 h. Chemical analysis were carried out as previously described (Bruneel et al., 2011). Briefly, the Fe(II) concentration was determined spectrophotometrically at 510 nm, the sulfate concentration was determined spectrophotometrically at 650 nm after precipitation with BaCl_2_ and stabilization with polyvinyl-pyrrolidone; arsenic speciation was performed by HPLC-ICP-MS using a Hamilton PRP-X100 column (250 × 4.6 mm) and a phosphate buffer (30 mM, pH = 8).

**Table 1:** **Physical and chemical characteristics of water samples**.

**Sampling date**	**Station**	**Physico-chemical parameters**			**Concentrations of the constituents of interest and redox As species**					
		**T (°C)**	**pH**	**Conductivity (μS/cm)**	**Oxygen concentration (mg/L)**	**Sulfate (mg/L)**	**Fe(II)(mg/L)**	**Total As (mg/L)**	**Proportion of As(III) (%)**	**Proportion of As(V) (%)**
30/06/2011	S1	14.8	3.15	4000	0.45	3837	1539	180.7	91.5	8.5
	COWG	15.5	3.23	3590	7.37	2798	1377	160.3	86.1	13.9
	CONF	16.8	6.07	1366	8.56	1081	5	0.02	25.6	74.4
24/01/2012	S1	13.7	3.79	3140	1.07	3479	955	154.9	75.9	24.1
	COWG	11.2	3.68	2640	8.51	2991	691	115.2	89.7	10.3
	CONF	7.6	5.14	971	9.57	806	8	10.3	87.0	13.0

### DNA extraction and pyrosequencing of bacterial 16S rRNA gene fragments

Triplicate genomic DNA was extracted from sediments using the UltraClean Soil DNA Isolation Kit in line with the manufacturer's recommendations (MoBio Laboratories Inc., Carlsbad, CA, USA). These triplicates were pooled before performing PCR amplification. All the genomic DNA samples extracted were stored at −20°C until further analysis. The 16S rRNA genes were amplified by PCR for multiplexed pyrosequencing using barcoded primers. The pairs of primers used, targeting the variable regions V3 to V5 of the 16S rRNA gene, were 343F (5′-AxxxTACGGRAGGCAGCAG-3′) (Liu et al., 2007) and 806R (5′-Bxxx GGACTACCAGGGTATCTAAT-3′) [(Teske and Sørensen, 2008), modified by Genoscreen (Lille, France)], where A and B stand for the two FLX Titanium adapters (A adapter sequence: 5′-CCATCTCATCCCTGCGTGTCTCCGAC-3′; B adapter sequence: 5′-CCTATCCCCTGTGTGCCTTGGCAGTC-3′) and xxx stands for the sample's specific bar-code sequence. Two PCR amplifications with two different barcodes were performed on each sample (n1 and n2). The reaction mixture contained 5–10 μL of DNA, 5 μL of 10X PCR buffer, 240 μM of dNTP, 1.5 mM of MgCl_2_, 1 μL of each of the primers (total amount: 10 μM), and 1U of taq polymerase. Sterile distilled water was added to obtain a final volume of 50 μL. After performing a denaturation step at 95°C for 10 min, 30 cycles were run as follows: 95°C for 30 s (denaturation), 50°C for 30 s (annealing) and 72°C for 1 min (extension), followed by 10 min at 72°C. Several PCR amplicons were pooled in the purification step because of the poor performances of the PCRs. Pyrosequencing was performed at Genoscreen (Lille, France) using a Roche 454 Life Sciences Genome Sequencer FLX Titanium sequencer.

### Processing of pyrosequencing data, diversity estimation, and taxonomic classification

The raw data generated by the 454 pyrosequencing procedure were processed and analyzed using the 1.32.1 version of mothur software (updated 10/16/13, http://www.mothur.org) (Schloss et al., 2009). These sequences were processed using the commands shhh.flows with the PyroNoise algorithm (Quince et al., 2009). The pre-processing of unaligned sequences also included removing short sequences (<200 bp), all sequences containing ambiguous characters as well as sequences with more than 8 homopolymers. We also removed any sequences that did not align over the same region. Identical sequences were then grouped together and representative sequences were aligned against the SILVA bacterial and archaeal reference database using the Needleman-Wunsch algorithm (Needleman and Wunsch, 1970). Chimeric sequences were detected and removed using the Chimera Uchime command (Edgar et al., 2011). A further pre-clustering screening step was performed in order to reduce the sequencing noise by clustering reads differing by only 1 base every 100 bases (Huse et al., 2010). The remaining high-quality reads were used to generate a distance matrix and were clustered into Operational Taxonomic Units (OTUs) obtained with a 97% cutoff using the “Average Neighbor” algorithm. The OTUs were then classified phylogenetically at the genus level using the naive Bayesian classifier (with a 80% confidence threshold) trained on the RDP's taxonomic outline and implemented in Mothur. Sequences were deposited in the National Center for Biotechnology Information (NCBI) Sequence Read Archive (SRA) under the accession number SRP068350. Nonparametric Chao1 and Shannon alpha diversity estimates as well as the coverage and rarefaction curves were calculated based on normalized data obtained on each sample with Mothur software.

### Fluorescence *in situ* hybridization (FISH) analysis

Sediments were fixed in freshly prepared 4% paraformaldehyde for 2 h 30 at 4°C, washed with phosphate buffered saline (PBS) and stored at −20°C in 50% PBS/ethanol solution within 8 h of being collected. Technical triplicates were performed with these samples. The FISH technique was performed on fixed samples as described by Hugenholtz et al. (2002) with slight modifications including incubation with bovine serum albumin (BSA) and dehydration with ethanol. Several methods (such as vortexing, sonication, manual scratching, the use of low melting agarose, lysozyme, and BSA) were compared for separating bacteria from the substratum, improving the hybridization, decreasing the background noise, and preventing cell loss. Optimum bacterial separation was obtained by performing sonication and the background noise was found to be lowest after performing BSA incubation. Optimization procedures were performed with a EUB338 probe (Amann et al., 1990). In order to detect any nonspecific binding of FISH-label probes, the samples were also hybridized with an antisense EUB338, the NON338 probe (Wallner et al., 1993). Very few or no probe-positive cells were detected using the negative control. After optimization, fixed samples were sonicated for 3 min, incubated for 20 min in BSA (0.4 mg/mL, Amresco, US), spread on 10-well Teflon coated glass slides (Menzel gläser, Germany) and dried for 10 min at 26°C. The samples were then dehydrated in 50, 80, and 90% ethanol solutions for 3 min each. Sediments were hybridized using the following specific fluorescent probes: EUB338 (Amann et al., 1990) hybridizes with most Bacteria, NON 338 (Wallner et al., 1993) for the negative control, TM1G0138, the specific probe targeting *Thiomonas* group 1 according to Hallberg et al. (2006), corresponding to Group II according to Bryan et al. (2009), and TM2G0138 with *Thiomonas* group 2 according to Hallberg et al. (2006), corresponding to Group I according to Bryan et al. (2009). The conditions of hybridization used were previously optimized by testing each probe against a collection of bacterial species kept at our laboratory (Hallberg et al., 2006). *In situ* hybridizations were performed at 46°C for 2 h in 0.9 M NaCl, 20 mM Tris-HCl at pH 8, 5 mM EDTA, 0.01% sodium dodecyl sulfate (SDS), 3–5 ng/μL of fluorescently labeled probes, and 30% formamide. Samples were washed at 48°C for 20 min in 20 mM Tris-HCl at pH 8, 0.01% SDS, 5 mM EDTA and 0.101 M NaCl, rinsed in distilled water, air dried, counterstained with 4′,6′-diamidino-2-phenylindole (DAPI, 1 μg/mL, 2 min) and mounted with Citifluor AF1 (Biovalley, France). Oligonucleotides labeled with the cyanine dye (Cy5) or Fluorescein isothiocyanate (FITC) at the 5′ end were purchased from Eurogentec (Belgium). Microscopic counting of at least 1000 DAPI-stained cells per sample was carried out on 20 microscopic fields in triplicate, (making a total number of 60 fields) using a confocal laser scanning microscope (LSM 510; Zeiss, Germany) and analyzed with the Image J program. Only cells doubly labeled with DAPI and EUB338 or triply labeled (i.e., DAPI, EUB338, and TM1G0138/TM2G0138) were counted.

### Protein extraction from the microbial community present in the reigous creek sediments

To harvest microbial cells, sediments were subjected to a Nycodenz® gradient. Ten 15-mL aliquots of the sediments were washed once in 15 mL of solution 1 [in g/L: Na_2_SO_4_.10H_2_O, 0.15; (NH_4_)_2_SO_4_, 0.45; KCl, 0.05; MgSO_4_.7H_2_0, 0.5; KH_2_PO_4_, 0.05; Ca(NO_3_)_2_.4H_2_O, 0.014] and agitated overnight at 4°C. After performing a 10-min decantation step to separate the sediments from the cells, 15 mL of supernatant were added without mixing to 35 mL of a 65% Nycodenz® solution (Axis-Shield, Oslo Norway) and centrifuged for 1 h at 10,000 g. The cellular fraction (the upper fraction) was removed and washed by adding 2 volumes of solution 1 and centrifuged for 15 min at 10,000 g at 4°C. Pellets were suspended in 600 μL of milliQ water supplemented with 1 μL Benzonase Nuclease (Sigma) and 4 μL of Protease Inhibitor Mix (GE Healthcare). Cell suspensions were disrupted by adding glass beads (1 g) and vortexing them 3 times for 1 min at 30-s intervals. Cellular debris were removed by performing two centrifugation runs, at 10,000 g for 5 min and at 16,000 g for 90 min. Protein concentrations were measured using the Qubit® Fluorometric Quantitation kit (Life technologies) in line with the manufacturer's instructions.

### Development of the liquid chromatography (LC) selected reaction monitoring (SRM) assay

#### Selection of targeted proteins and peptides

The targeted assay method used was specially developed for targeting arsenite oxidase (AioA/B), with RpoA serving as an ubiquitously expressed control protein. The AioA/B and RpoA protein sequences obtained from the sequenced genomes of 7 selected *Thiomonas* strains and the reconstructed Carn2 genome (Bertin et al., 2011; Arsène-Ploetze et al., 2012; Freel et al., 2015) were compared by aligning these sequences with Clustal W2 (http://www.ebi.ac.uk/Tools/msa/clustalw2/). Using the “Peptidemass” tool from Expasy (http://web.expasy.org/peptide_mass/), peptide sequences resulting from an *in silico* trypsin digestion having masses ranging between 500 and 1600 Da were selected. Peptides containing cysteine or methionine residues were ruled out. In order to test whether the peptides selected were proteotypic of the target protein, a search was conducted for their sequences in all the genomes available in October 2012 on the MicroScope (Microbial Genome Annotation and Analysis) Platform (https://www.genoscope.cns.fr/agc/microscope/home/index.php) using the pattern match search tool, taking into account the fact that Q, E and K, or N and D or I and L have non distinctive masses. Only peptides that were specific to the proteins present in *Thiomonas* strains were selected manually. Fifty peptides were selected in this way and synthesized in the form of crude isotopically-labeled standard peptides (PEPotec Peptides, Thermo Fisher Scientific, Ulm, Germany) before being spiked into protein extracts obtained from four isolated *Thiomonas* strains grown at our laboratory (*Thiomonas* sp. CB2, CB1, CB3, and CB6) in the absence and in the presence of arsenite. Sixteen peptides giving satisfactory LC-MS signals were used to perform the assay on sediment samples (Supplementary Tables 1,2).

#### Selection and optimization of transitions

The Skyline open-source software program (MacLean et al., 2010) was used to set up the spectral library and generate the list of transitions. An MS/MS spectral library was acquired on the mixture of 16 heavy labeled synthetic peptides, as described in Supplementary Data. At least three transitions were monitored per peptide in order to identify the peptide, and the quantification was performed only on transitions showing no interferences. The individual collision energies were also optimized experimentally using the heavy standard peptide mixture injected into microLC-SRM and testing nine values centered on the theoretical value provided by the supplier. A complete list of the transitions recorded is presented in Supplementary Table 1, along with the corresponding retention times and optimized collision energies.

#### General experimental workflow

After the reduction and alkylation steps, protein samples were digested with trypsin. The tryptic peptides obtained were desalted by performing Solid Phase Extraction (SPE) before microLC-SRM analysis. Twenty-four samples of isolated bacterial strains [eight samples of four strains (*Thiomonas* sp. CB2, CB1, CB3, and CB6)] grown *in vitro* with and without arsenite were prepared in triplicate and referred to here as “biological triplicates.” Biological triplicates were randomly analyzed in injection triplicates (5 μg injected). Five randomly chosen bacterial strain samples serving as quality control (QC) samples were pooled, prepared as described below, and injected (10 μg) throughout the experiment. Sediment samples were replicated whenever possible, but in a few cases, this was not possible because too little protein material was available.

#### Sample preparation and protein digestion

Samples of bacterial strains were distributed randomly into five batches, which were extemporaneously digested before LC-SRM analysis. Fifty micrograms of each sample were denatured with 100 μL of 8 M urea and 0.1 M NH_4_HCO_3_ and spiked with a mixture of crude heavy labeled peptides in order to obtain signal intensity ratios close to one between the light and heavy transitions (the relative volumes of each heavy peptide were determined experimentally in order to obtain a concentration-balanced standard peptide mixture). Reduction, alkylation, liquid trypsin digestion, and sample desalting were performed as described in detail in Supplementary Data.

#### Microlc-SRM parameters

LC-SRM analyses were performed on a Dionex Ultimate 3000 MicroLC coupled to a TSQ Vantage triple-quadrupole (Thermo Scientific). The system was entirely controlled by Xcalibur 2.1 (Thermo Scientific). Peptides were first trapped on a μ-Precolumn (Zorbax C18 stable bond, 5 μm, 1.0 × 17 mm, Agilent Technologies, Palo Alto, CA, USA) before being separated on a Thermo Scientific Acclaim capillary column (PepMap100, C18, 300 μm, 15 cm, 3 μm, 100 Å). The solvent system used here consisted of 2% acetonitrile and 0.1% HCOOH in water (v/v/v, solvent A) and 2% water and 0.1% HCOOH in ACN (v/v/v, solvent B). Trapping was performed for 5 min at 30 μL/min with solvent A. Elution was performed at a flow rate of 5 μL/min, using a 1–25% gradient (solvent B) for 80 min at 35°C. Gradient was followed by a 5-min step with 80% B before returning to the initial conditions. The mass spectrometer was equipped with an HESI source operating in the positive mode. Classical instrument operating parameters were used. The isolation width adopted with both Q1 and Q3 was set at 0.7 m/z unit. Scheduled SRM was used for data acquisition purposes: each transition was monitored during a 10- and 15-min time window centered at previously determined peptide retention times in the case of bacterial strains and sediment samples, respectively. Cycle time was set at 2.5 s and minimum dwell times were set at 25 ms and 17 ms, respectively.

#### Data interpretation

The Skyline application was used to display the LC-SRM data and to perform peak picking and the integration of transition peak areas. Area intensity ratios between the heavy and light forms of each peptide were checked manually. Quality control was performed on the experiment by examining the stability of the SRM-MS signal obtained with QC samples. The QC sample was analyzed 13 times in all during the experiment. Relative peptide quantification and testing for differential peptide expression were performed using the MSstats R-package (Chang et al., 2012). Differences in protein abundance with a *p*-value of < 0.05 and a difference greater than two-fold were taken to be statistically significant.

## Results and discussion

### Spatio-temporal quantification of *Thiomonas* strains using pyrosequencing and fluorescence *in situ* hybridization (FISH) methods

Quantification of *Thiomonas* strains was performed at three different locations along the Reigous creek (Source, COWG, and CONF) and at two different time points (a summer point in June 2011 and a winter point in January 2012). First, the presence and abundance of *Thiomonas* bacteria was assessed by performing 16S rRNA gene sequencing on DNA extracted from sediment samples.

**Figure 1:**
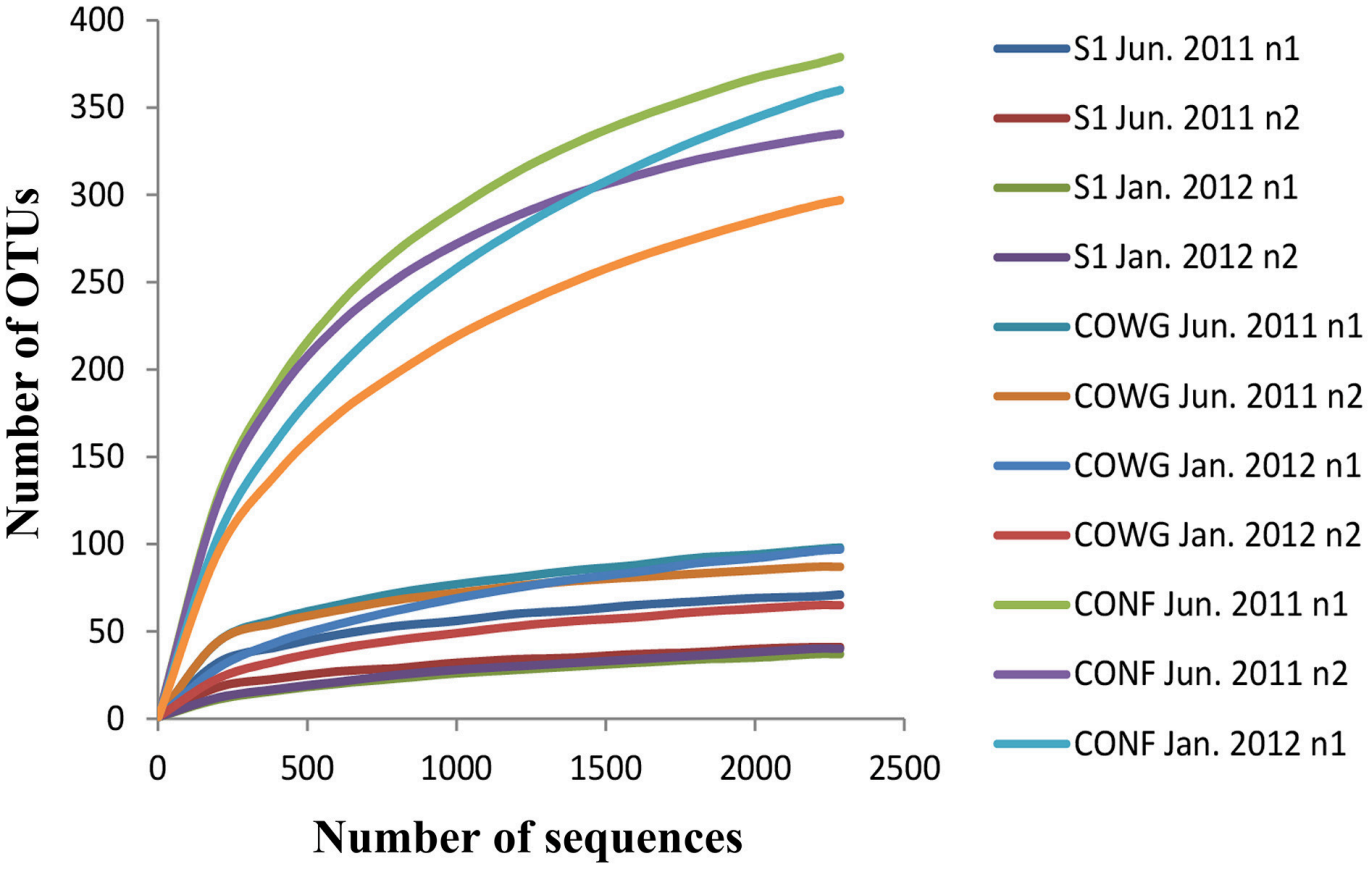
**Rarefaction curves of the bacterial 16S rRNA gene sequences obtained using pyrosequencing methods on sediments from the Reigous creek based on OTUs calculated at 97% identity in the normalized dataset**. The total number of sequences analyzed is plotted against the number of OTUs observed in the same library.

A total number of 277,871 sequence reads with a mean length of around 400 bp were generated in a single 454 pyrosequencing run from the 12 independent 16S rRNA gene libraries (six samples in duplicate). After trimming and processing the data with Mothur, there were 143,123 remaining reads (average mean length 245 bp). In order to obtain comparable data between the sampling sites and compare them, the same number of sequences per sample was obtained using random resampling methods (2285 sequences, making a total number of 27,420) and their clustering led to the identification of 913 OTUs defined at 97% identity (Supplementary Table 3). The fact that the rarefaction curves tended to reach an asymptote with most of the samples (Figure 1) suggests that the majority of the bacterial phylotypes present in each sample were identified. This was confirmed by the very high Good's coverage of all the duplicates (ranging from 95 to 99%, Supplementary Table 3), which gives an estimate of the sampling completeness based on a probability calculation with randomly selected amplicon sequences. The non-parametric estimator Chao1 varied from one site to another along the Reigous: the lowest OTU richness (50–88) was observed in S1 and the highest levels (365–445) were recorded in CONF (Supplementary Table 3). The same trend was observed with the Shannon index, which showed the lowest levels of diversity (0.78–2.86) in S1 and the highest levels in CONF (4.69–5.40) (Supplementary Table 3).

Fourteen bacterial phyla were recovered from our samples. The majority of the bacterial sequences (57%) belonged to the Proteobacteria phylum (Supplementary Table 4). This phylum was dominated by *Gallionellales, Acidithiobacillales*, and *Burkholderiales*, (Supplementary Table 5), in line with previous analyses (Bruneel et al., 2011; Volant et al., 2014). Among the *Burkholderiales, Thiomonas* accounted for only a small proportion of the OTUs identified. At a confidence threshold of 80%, 469 sequences in all (228 and 241 sequences in the first and second duplicates n1 and n2, respectively) belonged to OTUs which could be assigned to the *Thiomonas* genus, amounting to 1.71% of the normalized data set (data not shown). However, to gain further information about this genus, a taxonomic classification was performed directly on the sequences obtained in the whole data set (Table 2). In this way, 1881 sequences (1008 and 873 sequences with n1 and n2, respectively) were assigned to the *Thiomonas* genus, amounting to 1.3% of the total number of sequences. These sequences were assigned to various strains belonging to the phylogenetic Group I (*Thiomonas* Ynys3 and *Thiomonas intermedia*) and Group II (*Thiomonas* PK44, *Thiomonas arsenivorans, Thiomonas cuprina*, and *Thiomonas* sp. DM-Cd4) according to Bryan et al. (2009) or to unclassified *Thiomonas* species (Table 2). All in all, the high-throughput sequencing methods used in this study brought to light sequences corresponding to the *Thiomonas* genus and confirmed the persistence of this genus in the Reigous creek since it was first isolated in 2001 (Bruneel et al., 2003). Sequences affiliated to the *Thiomonas* genus accounted for only a small proportion of the total bacterial community (<2% of the total sequences). However, due to the technical bias inherent to this technique and the small size of the fragments generated, the pyrosequencing procedures used here cannot be regarded as a quantitative method.

**Table 2:** **Number of sequences assigned to the genus ***Thiomonas*** based on pyrosequencing analyses**.

**Taxon**	**Group**	**Total n1**	**S1 Jun. 2011 n1**	**S1 Jan. 2012 n1**	**COWG Jun. 2011 n1**	**COWG Jan. 2012 n1**	**CONF Jun. 2011 n1**	**CONF Jan. 2012 n1**	**Total n2**	**S1 Jun. 2011 n2**	**S1 Jan. 2012 n2**	**COWG Jun. 2011 n2**	**COWG Jan. 2012 n2**	**CONF Jun. 2011 n2**	**CONF Jan. 2012 n2**
Total		1008	190	84	105	114	274	241	873	42	115	165	118	98	335
*Tm*. PK44	II	1	0	0	0	0	0	0	573	40	115	150	118	19	131
*Tm*. Ynys3	I	4	0	0	0	0	0	1	1	0	0	0	0	0	1
*Tm. arsenivorans*	II	3	0	0	0	0	0	3	15	0	0	0	0	0	15
*Tm. cuprina*	II	25	0	0	0	0	0	0	18	0	0	0	0	18	0
*Tm. intermedia*	I	184	7	1	5	0	11	1	8	1	0	7	0	0	0
*Tm*. sp. DM Cd4	II	979	0	0	2	1	104	52	99	0	0	4	0	52	43
Unclassified	?	820	183	83	98	113	159	184	159	1	0	4	0	9	145

**Figure 2:**
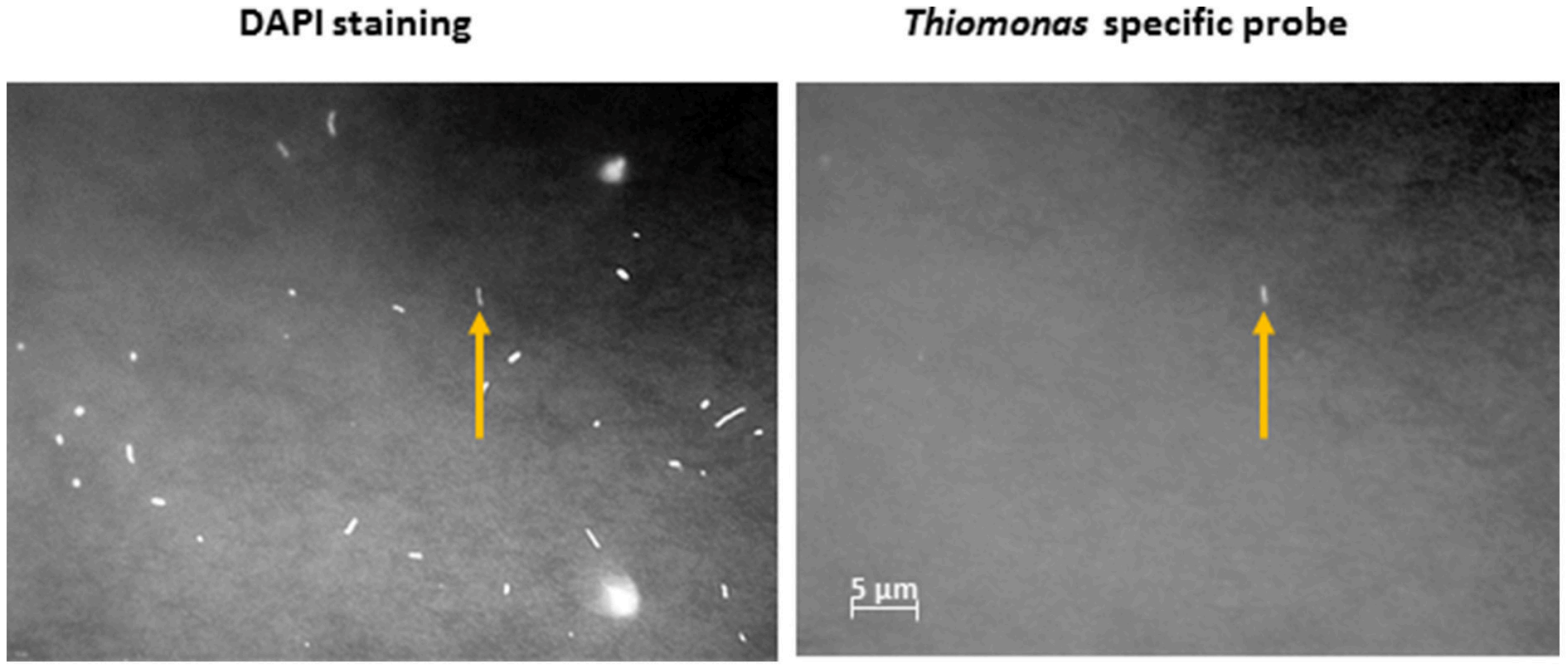
**FISH micrographs of cells labeled with the oligonucleotide probe TM1G0138 detecting Group II ***Thiomonas*** bacteria in sediment from the COWG site (collected in January 2012)**. Most of the bacteria show DAPI staining (**left** panel), and cells targeted with TM1G0138 are labeled with Cy5 (**right** panel). Scale bars = 5 μm. The arrow indicates one *Thiomonas* cell.

**Figure 3:**
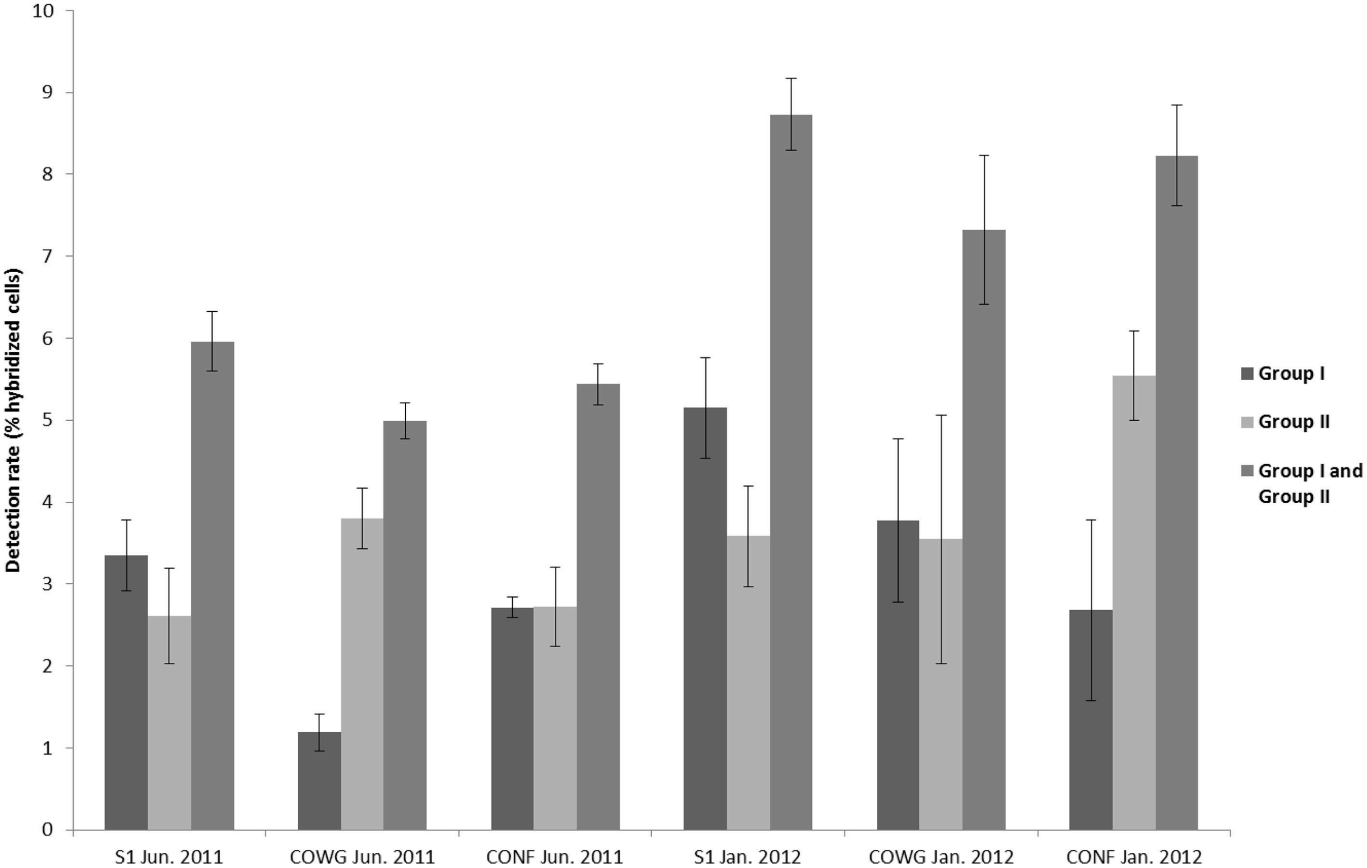
**Estimated proportions of ***Thiomonas*** bacteria hybridized with the oligonucleotide probe TM1G0138 in the case of Group II according to Bryan et al. (2009), with the oligonucleotide probe TM2G0138 in that of Group I according to Bryan et al. (2009) and the proportions of ***Thiomonas*** Groups I and II combined**. Error bars correspond to standard deviations calculated on technical triplicates.

In order to gain further insights into the actual abundance of the *Thiomonas* belonging to each phylogenetic group, a microscopic analysis of samples labeled with FISH probes was therefore performed using two probes targeting either *Thiomonas* Group I or *Thiomonas* Group II (Hallberg et al., 2006; Bryan et al., 2009) (see Section Materials and Methods). In parallel, the number of total bacteria was also determined. The results obtained using this approach showed that 79.6–90.1% of the DAPI-stained cells were stained with the bacteria-specific probe EUB338: the DAPI-stained cells included some fungal and minor groups of bacteria that did not hybridize with EUB338 (Figure 2). *Thiomonas* specific probes indicated that phylogenetic Groups I and II were present both in June 2011 and January 2012 at all the sampling sites along the Reigous creek (S1, COWG, and CONF) (Figures 2, 3). The abundances ranged from 2.6± 0.4% to 5.5 ± 1.1% in the case of Group II and 1.2 ± 0.4% to 5.1% ± 0.6% in that of Group I and 5 to 8.7% in Groups I and II combined (Figure 3). These levels of abundance are higher than those obtained using pyrosequencing methods, but FISH is generally held to be a more suitable method for counting microorganisms than molecular methods based on PCR amplification (Amann et al., 2001; Wagner and Haider, 2012). Although both approaches showed the presence of *Thiomonas* Group I and Group II in summer 2011 and winter 2012 along the whole Reigous creek, these bacteria constituted only a small proportion of the total bacterial community (<9%). These results corroborate the findings made in previous studies on Carnoulès water and sediments (Table 3), which showed that *Thiomonas* were sustainably present at this site. In addition, the present study shows that several phylogenetic groups of *Thiomonas* constitute a minor but stable fractions of the population observed for more than a decade at various sampling points along the Reigous creek. Interestingly, despite the low abundance of these populations, this genus has been thought to play a crucial role in the efficient remediation processes observed in this creek by favoring the oxidation of As(III) into the less mobile and less toxic form As(V) and its co-precipitation with Fe(III) (Casiot et al., 2003; Morin et al., 2003; Bertin et al., 2011). Therefore, to test whether these *Thiomonas* spp. are actually active and express key enzymes, a targeted proteomic approach was developed in order to detect the presence of *Thiomonas* arsenite oxidases.

**Table 3:** **Studies showing the presence of ***Thiomonas*** in the Reigous creek**.

**Date**	**Sampling sites**	**Type of *Thiomonas***	**Abundance**	**Approach used**	**References**
Every 3–4 weeks from June 2001 to August 2002	Four locations along the first 150 m (water) including S1 et COWG	Group I (B1; B2; B3)	≈0,5%	Cultivation	Bruneel et al., 2003
October 2002 and January 2003	S1, COWA and COWG (water)	None detected	0	Terminal-restriction fragment length polymorphism (T-RFLP) and 16S rRNA gene library analyses	Bruneel et al., 2006
April 2006	COWG (water and sediments)	Group II	6% sediments, 3% water	16S rRNA gene library analyses; metaproteomic	Bruneel et al., 2011
May 2007	COWG (sediments)	Group II	Not quantified	Metagenomic, metaproteomic	Bertin et al., 2011
November 2009	Sediment at S1, COWG, GAL and CONF	Group II (X19)	Not quantified	Cultivation	Delavat et al., 2012
From November 2007 to March 2010	Water at S5 (inside the tailing), S1, COWG, GAL and CONF	?	<1%	Pyrosequencing	Volant et al., 2014
June 2011	Sediments at S1, COWG, CONF	Group I and II	<1%	Pyrosequencing	This study
			5–6%	FISH	
January 2012	Sediments at S1, COWG, CONF	Group I and II	<1%	Pyrosequencing	
			7–9%	FISH	

### Spatio-temporal detection of *Thiomonas* arsenite oxidase using a targeted proteomics approach based on liquid chromatography selected reaction monitoring mass spectrometry (LC-SRM)

The activity of *Thiomonas* spp. in the Carnoulès ecosystem was previously reported in two general metaproteomic studies (Bertin et al., 2011; Bruneel et al., 2011). In the first study (Bruneel et al., 2011), the activity of *Thiomonas* was suggested at the COWG sampling point, as the GroEL and 50S ribosomal L1 proteins were identified using a 2-D gel based metaproteomic approach (Table 3). In a second SDS-PAGE-based metaproteomic study (Bertin et al., 2011), 89 proteins including 3 arsenite oxidases (AioA/B) originating from *Thiomonas*-like Carn2 cells were identified at the COWG sampling point, which showed for the first time that these bacteria indeed expressed these enzymes which play a crucial role in the *in situ* remediation processes (Table 3). In order to test whether the minority population detected at several sampling sites and time points always expressed the AioA/B proteins, we developed a targeted method based on the use of an LC-SRM approach. Since this approach shows the best sensitivity and specificity among the various proteomic approaches available, we attempted to identify the *Thiomonas* arsenite oxidase proteins present in the complex protein mixture extracted from the Reigous creek sediment community without performing any sample fractionation (Arsène-Ploetze et al., 2012, 2015).

Three proteins were targeted for this purpose: AioA and AioB, the two subunits of arsenite oxydase, and RpoA, a ubiquitously expressed protein which was selected as a control protein. RpoA also has the advantage of including peptides which can be used to discriminate between *Thiomonas* Groups I and II. The success of a SRM-based targeted assay depends on choosing the most appropriate proteotypic peptides for use as specific tracers of each of the proteins of interest (Gallien et al., 2011; Arsène-Ploetze et al., 2012, 2015). Extensive *in silico* and experimental studies were therefore performed in order to be able to discriminate with the greater possible sensitivity between the *Thiomonas* Group I and Group II proteins present (Supplementary Table 2). *In silico* screening for uniqueness was performed on all the genomes available on the MicroScope (Microbial Genome Annotation and Analysis) Platform. Only peptides that were specific to the proteins from *Thiomonas* Groups I and/or II were manually validated. Heavy isotopically labeled peptides were then synthesized for the peptides selected and used to determine the best transitions, i.e., the number and types of fragments to be measured for each of the peptides selected, and to optimize the MS instrument parameters. Sixteen peptides showing promising MS-signals were used to optimize 136 specific light/heavy transitions (Supplementary Tables 1,2). A concentration-balanced mixture of heavy labeled peptides was prepared in order to obtain homogeneous signals in the case of all the peptides, and the samples were spiked with this mixture in order to be able to quantify the endogenous peptides of interest by calculating heavy/light ratios.

**Figure 4:**
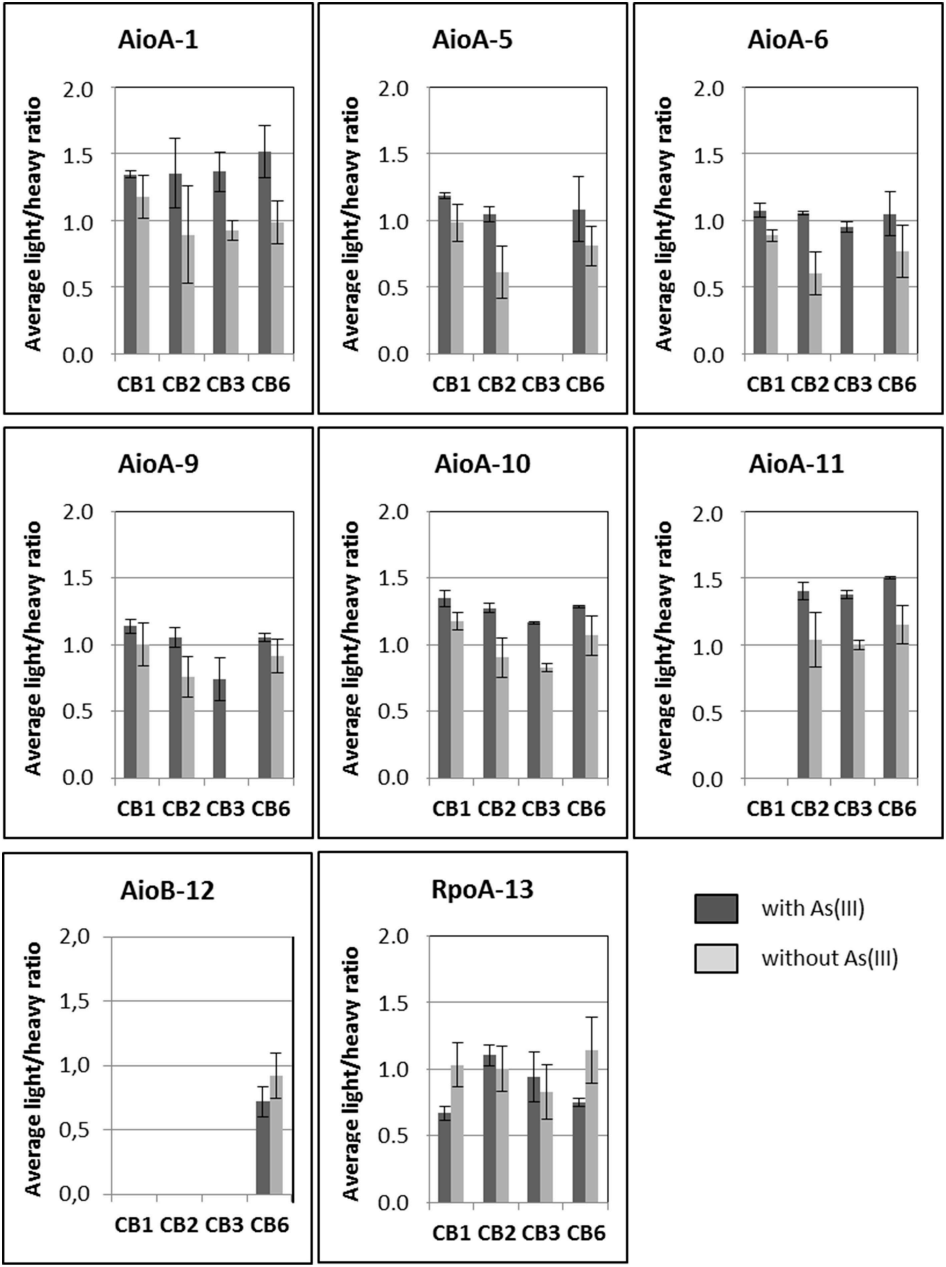
**Quantification of arsenite oxidase specific peptides (average light/heavy ratios calculated from biological and injection triplicates) in ***Thiomonas*** CB1, CB2, CB3, and CB6 cells grown in the absence or presence of 1.3 mM As(III) in m126 medium**. Biological triplicates and technical triplicates are plotted. Error bars correspond to standard deviations calculated for the nine replicate values (biological and technical replicates).

First, a test experiment was set up to detect the peptides of interest in total cell lysates obtained from four *Thiomonas* strains, CB1, CB2, CB3, and CB6 (Group I *Thiomonas*), grown *in vitro* in the presence or absence of arsenite. This experiment was performed on both biological triplicates and injection triplicates. Eight of the 16 peptides were detected and quantified, including six peptides specific to AioA (peptides 1, 5, 6, 9, 10, and 11), one specific to AioB (peptide 12) and one specific to RpoA (peptide 13) (Table 4, Figure 4 and Supplementary Table 6). Comparisons between the light/heavy ratios led to the conclusion that the levels of expression of AioA were higher when CB1, CB2, CB3, and CB6 were grown in the presence than in the absence of As(III) (Figure 4 and Supplementary Table 6). These results were in agreement with previous findings showing that the expression of this protein is induced in *Thiomonas* cells in the presence of arsenite (Freel et al., 2015). The AioB peptide (peptide 12) was not detected in CB1, CB2, or CB3 because the signal recorded was below this peptide's quantification limit. Peptides 2 and 15, which are specific to Group II *Thiomonas*, were naturally not detected in the experiments performed on proteins extracted from Group I *Thiomonas* (Table 4). However, since these two peptides have been identified in previous global metaproteomic studies they were included in the analysis of sediment samples (Bertin et al., 2011) (Table 4).

**Table 4:** **Peptides used to detect AioA/B and RpoA from ***Thiomonas*** in protein extracts from cell cultures of isolated strains and from the community inhabiting the Reigous creek sediments at various points in space and time**.

**Targeted protein**	**Peptide number**	**Sequence**	**Specificity Tm.I/Tm.II^#^**	**Global proteomics 2007 study (Bertin et al., 2011)**	**Cell cultures**	**2007 COWG**	**2009 COWG**	**2009 COWG**	**2011 COWG**	**2012 COWG**
AioA	1	ACVVNQGLSSTR	Tm.I/Tm.II	X	CB1, CB2, CB3, CB6	X	–	–	–	–
	2	IQIFPAK	Tm.II (Carn2_0821 only)^*^	X	–	–	–	–	–	–
	3	DFIANHTEGFDAAVK	Tm.II (Carn2_1469 only)^*^	–	–	–	–	–	–	–
	4	DFIANHTEGFEAAVK	Tm.II (Carn2_0821, Carn2_1330)	–	–	–	–	–	–	–
	5	FWINNGR	Tm.I/Tm.II	X	CB1, CB2, CB6	X	–	–	–	–
	6	YPAADFPIPR	Tm.I/Tm.II	X	CB1, CB2, CB3, CB6	X	X	X	X	X
	7	YPASSVPIPR	*T. intermedia* K12 (Tm.I)	–	–	–	–	–	–	–
	8	DFIAQHTEGFEAAVK	Tm.I	–	–	–	–	–	–	–
	9	IQVFPAK	Tm.I/Tm.II	–	CB1, CB2, CB3, CB6	X	–	–	–	–
	10	ITGVPVAQIK	Tm.I/Tm.II	X	CB1, CB2, CB3, CB6	X	X	X	X	X
	11	LIFTGIQTPTVR	Tm.I/Tm.II	X	CB2, CB3, CB6	X	X	X	X	X
AioB	12	AVAVTGLIYGR	Tm.I/Tm.II	X	CB1, CB6	X	X	X	X	X
RpoA	13	SETELLK	Tm.I/Tm.II	–	CB1, CB2, CB3, CB6	X	–	–	–	–
	14	SIGHIVLDASFSPVR	Tm.I	–	–	–	–	–	–	–
	15	SIQVEALGPLR	Tm.II	X	–	–	X	X	X	X
	16	SIQVESLGHNR	Tm.I	–	–	–	–	–	–	–

# *Tm.I and Tm.II: specific to Thiomonas Group I and Group II, respectively, according to Bryan et al. (2009)*.

* *This peptide is specific to one of the three AioA proteins found to exist in the Thiomonas-like CARN2, Group II (Bertin et al., 2011)*.

**Figure 5:**
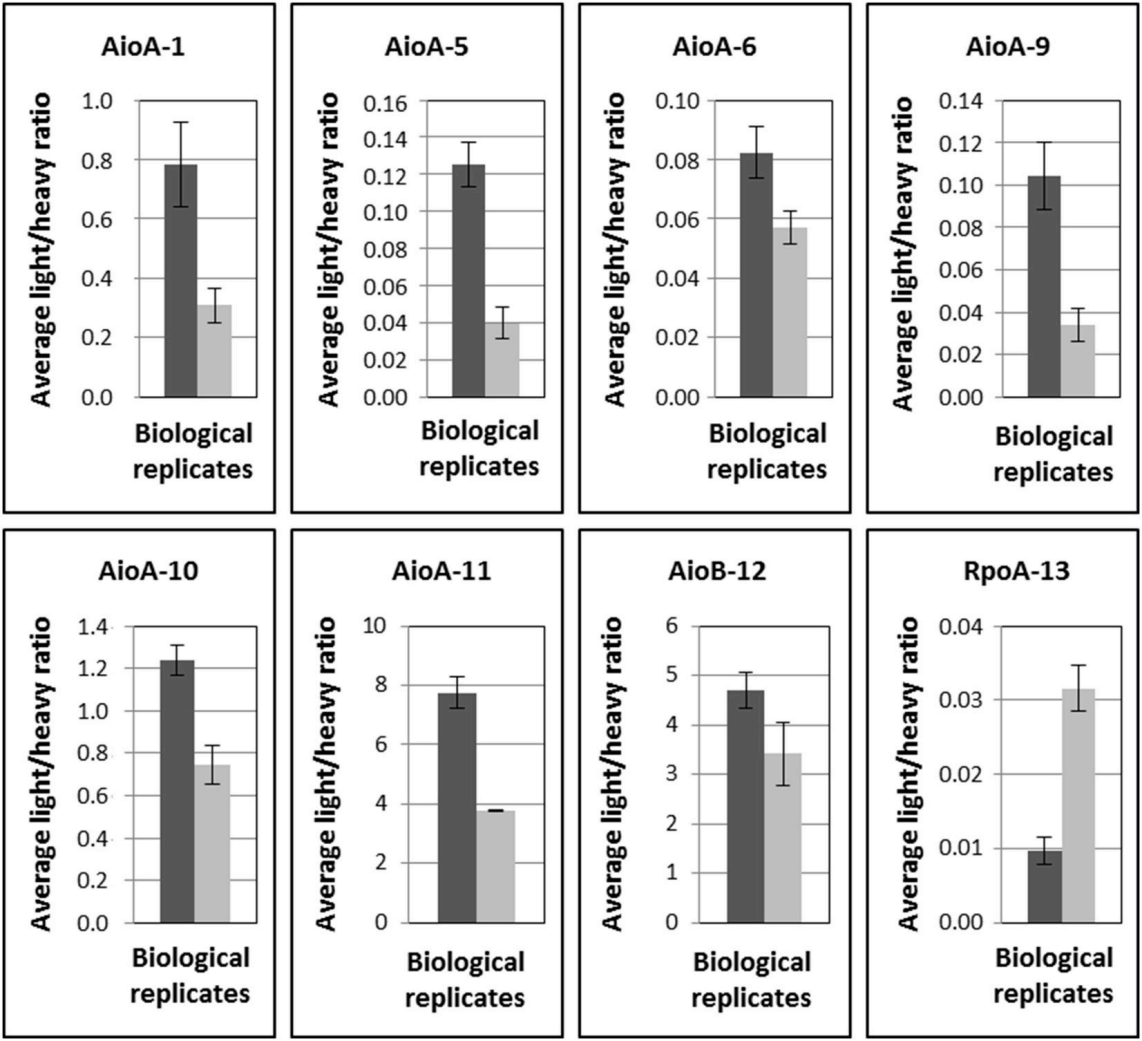
**Detection and quantification (average light/heavy ratios calculated from triplicate injections applied to two biological replicates) of ***Thiomonas*** arsenite oxidase and RpoA specific peptides in sediments of the Reigous creek sampled in 2007 (Bertin et al., 2011)**. Data on two biological replicates are plotted. Error bars correspond to standard deviations calculated for the technical replicates for each biological replicate.

The optimized LC-SRM assay was then tested on a metaproteomic test sample extracted in 2007 at the COWG site, in which the arsenite oxidases were previously detected (Bertin et al., 2011). This experiment was performed with duplicate biological replicates and 2 or 3 injection replicates (Figure 5). Surprisingly, peptides 2 and 15, which are specific to Group II *Thiomonas*, were not detected although they were identified in the previous overall analysis performed on the same samples (Bertin et al., 2011). This suggests that these proteins may be present in very low levels, which would mean the peptides were just below the detection limits. Eight peptides which were not specific to either Group I or II *Thiomonas* (peptides 1, 5, 6, 9, 10, 11, 12, and 13) were detected in this metaproteomic test sample (Table 4, Figure 5), which confirms that *Thiomonas* were active in these sediments and expressed the arsenite oxidases.

Lastly, the optimized LC-SRM assay was applied in order to detect the target proteins present in sediments extracted at different points in time and space, in June 2011 and January 2012 at the same sampling points as those at which the FISH and pyrosequencing analyses were performed. Large enough amounts of protein material could be extracted only from the samples originating from the COWG site in 2011 and 2012. The nature of the sediments obviously differed between COWG and CONF: those sampled at CONF were brown and their granulometry also differed from that of the beige sediments extracted at COWG, which may also have affected the Nycodenz-based cell extraction procedure. Additional samples, which had been extracted in 2009 at the CONF and COWG sites, were therefore included, in which *Thiomonas* cells had been detected and the physical and chemical characteristics of the water were available (Delavat et al., 2012; Volant et al., 2014) (Table 3). None of these samples contained sufficiently large amounts of proteins to be able to perform a reliable quantification assay with appropriate numbers of replicates, but they were nevertheless used to perform a qualitative analysis, i.e., to detect the proteins of interest. One peptide specific to RpoA (peptide 15), which is uniquely specific to Group II *Thiomonas*, was detected and thus confirmed the stable presence of *Thiomonas* strains belonging to Group II at the COWG and at CONF sites (Table 4 and Supplementary Figure 1). As no peptides specific to Group I *Thiomonas* could be detected on the contrary, their activity could not be confirmed. Peptides 6, 10, 11 (AioA), and 12 (AioB), all of which are common to both Groups I and II, were detected in samples obtained in 2009, 2011 and 2012 at COWG and in 2009 at CONF, where it was previously established that the arsenic concentration in the water was below 6 mg/L (Volant et al., 2014). The results obtained in the present study show that the *Thiomonas* arsenite oxidase is synthesized at three different places, at the source and downstream of the Reigous creek, and even at a point where the As concentration is low (Table 4 and Supplementary Figure 1).

Generally speaking, it was observed here that the levels of Group I *Thiomonas* varied with time (1% at COWG in 2011 as compared with 3.5% in 2012) and from one place to another (between S1, COWG and CONF in 2011), whereas the levels of the Group II *Thiomonas* are quite stable from one time and one place to another (except for CONF 2012). The physicochemical parameters, especially the pH, the temperature, the redox potential and the arsenic concentration, as well as the diversity and the spatiotemporal distribution of the bacterial communities were found to vary along the creek and with time (Tables 1, 4) (Egal et al., 2010; Volant et al., 2014). Despite these variations, *Thiomonas* strains were ubiquitously present in a fairly wide range of concentrations in the Reigous creek. In addition arsenite oxidase was detected in the 2007, 2009, 2011, and 2012 samples. Therefore, the present data show that the arsenite oxidase enzyme is expressed at each time point and at each of the locations tested, which suggests that these bacteria are continuously active and efficiently enhance the process of arsenic remediation at work in the Reigous creek. Abiotic Fe(II) oxidation without the need for bacterial catalysis and subsequent Fe and As precipitation in the form of As-rich ferrihydrite predominate in waters where the pH level is practically neutral (Singer and Stumm, 1970), i.e., downstream the confluence between the Reigous and Amous (Casiot et al., 2005, 2009). In contrast, the abiotic processes involved in the attenuation of metals [oxidation of Fe(II) into Fe(III) and As(III) into As(V)] occur slowly at acidic pH, i.e., the conditions observed in the Reigous creek (Table 1). Since the bacterial activities observed accelerate these processes, they play an essential role in the remediation occurring at this site. As(III) present here can be removed from the dissolved phase without undergoing an oxidation step, by forming tooéléite (Morin et al., 2003) for example, or through adsorption onto schwertmannite (Maillot et al., 2013). Nevertheless, the oxidation of As(III) into As(V) improves the efficiency of the remediation process because the concentration of dissolved As(V) in equilibrium with Schwertmannite-As(V) is lower than that of dissolved As(III) in equilibrum with Schwertmannite-As(III) (Maillot et al., 2013). Besides the arsenic oxidation, the methylation of arsenic may also contribute to the remediation process, since genes involved in such a process were previously detected in metagenomic data (Bertin et al., 2011). Furthermore, this methylation capability has been evidenced in microcosm experiments carried out with sediments from the Amous River (Héry et al., 2014). However, methylated arsenic species were not detected in the water from Reigous Creek or Amous River during the long-term monitoring of the Carnoulès site (Casiot et al., 2009; Egal et al., 2010), showing that this process does not seem to play a particularly important role *in situ*.

In conclusion, the optimized SRM assay developed in this study and applied without performing any sample fractionation to highly complex protein mixtures extracted from a microbial community proved to be sensitive enough to detect the AioA/B expressed by minor members of the bacterial community amounting to < 9% of the community. This detection efficiency suggests that the arsenite oxidase protein was very strongly expressed by these *Thiomonas*. The expression of arsenite oxidase was previously reported to occur at one of the present study sites (COWG) at one time point (May 2007) (Bertin et al., 2011). In the present study, it was established that several *Thiomonas* strains present in several places expressed arsenite oxidase at different time points, which indicates that the arsenite oxidation activity crucially involved in the natural attenuation processes probably occurred permanently in the Reigous creek from 2007 to 2012.

## Author contributions

OB, MH, AV, ChC, and FA designed the research project; AH, OB, CoC, AD, JF, MH, ChC, and FA performed the research; AH, OB, CoC, AD, MH, ChC, and FA wrote the paper.

### Conflict of interest statement

The authors declare that the research was conducted in the absence of any commercial or financial relationships that could be construed as a potential conflict of interest.

## Supplementary material

Supplementary Figure 1 **Detection of the AioA/B and RpoA peptides using the SRM assay**. Chromatograms were extracted with Skyline (MacLean et al., 2010). Two locations **(A,B)** were tested at three different time points **(B–D)**. **(A)** CONF 2009; **(B)** COWG 2009; **(C)** COWG 2011; **(D)** COWG 2012. Each transition is indicated by one color. On the right, in the peak area windows, a dot *p*-value close to 1 indicates the existence of a very good correlation between the transitions and relative fragment intensities of the endogenous peptides and the reference library spectra.

Supplementary Table 1 **Peptides used in this study and their measured transitions, retention times, and optimized collision energies**.

Supplementary Table 2 **Peptides designed to detect AioA/B and RpoA of Thiomonas strains**. Theoretical presence of the peptides indicated by a "X" was supposed based on the genomic data.

Supplementary Table 3 **Estimated OTU richness, diversity indices and estimated sample coverage for each 16S rRNA libraries using pyrosequencing analysis**. Results are presented for the normalized data, randomly resampled to have an equal sample size.

Supplementary Table 4 **Distribution of normalized sequences of bacterial 16S rRNA genes from each samples assigned to different phyla**.

Supplementary Table 5 **Distribution of normalized sequences of bacterial 16S rRNA genes of the phylum of Proteobacteria assigned to different orders**.

Supplementary Table 6 **Detection of AioA, AioB, and RpoA in Thiomonas after growth in m126 medium in the presence or in the absence of As(III)**.
