# Supplementary material for: Spatio-Temporal Detection of the Thiomonas Population and the Thiomonas Arsenite Oxidase Involved in Natural Arsenite Attenuation Processes in the Carnoulès Acid Mine Drainage
Source: Front Cell Dev Biol. 2016 Feb 1;4:3. doi: 10.3389/fcell.2016.00003 (PMC4734075; doi:10.3389/fcell.2016.00003)

Peptide6-**AioA** -YPAADFPIP**R**

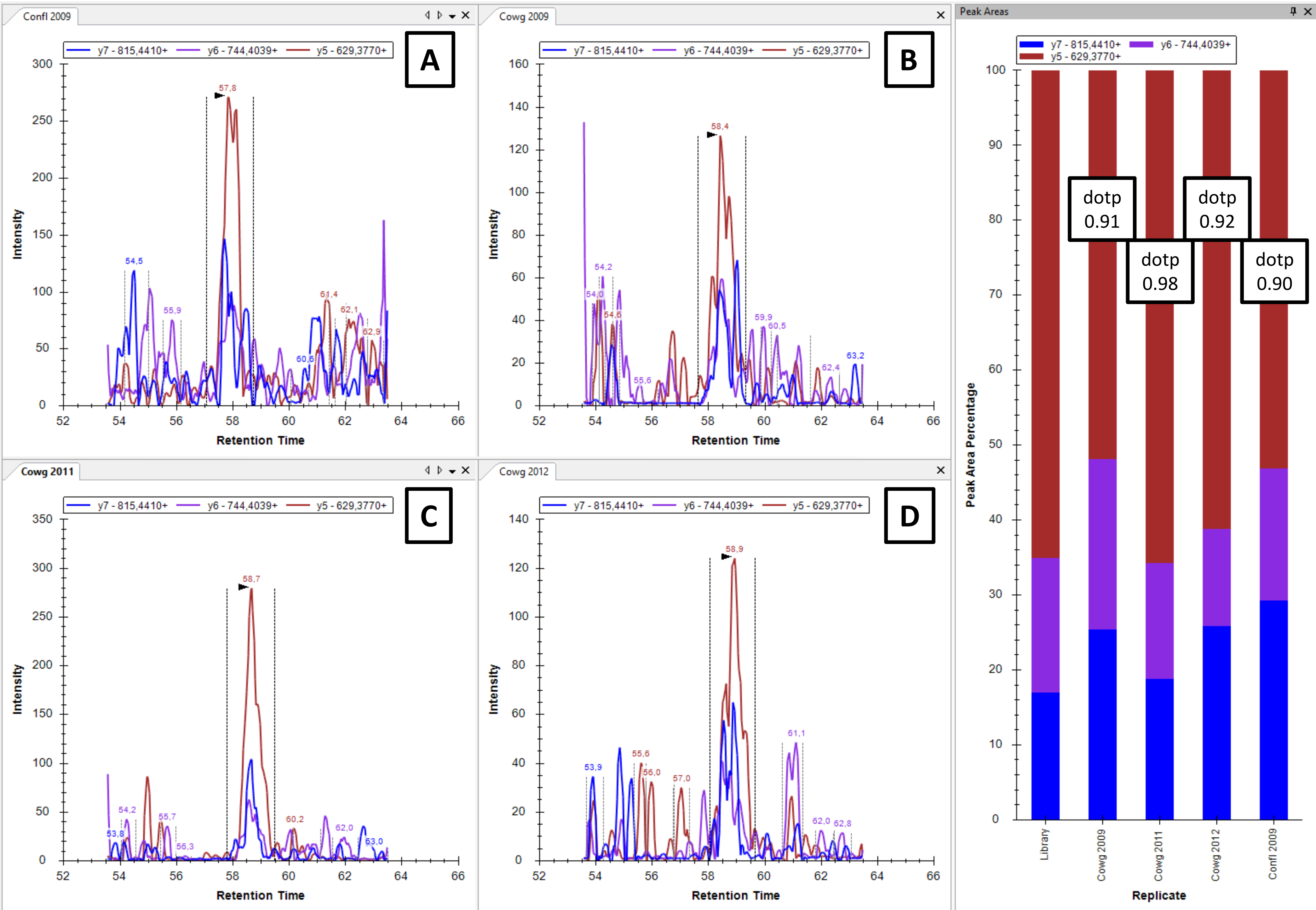

Peptide10-**AioA**-ITGVPVAQIK

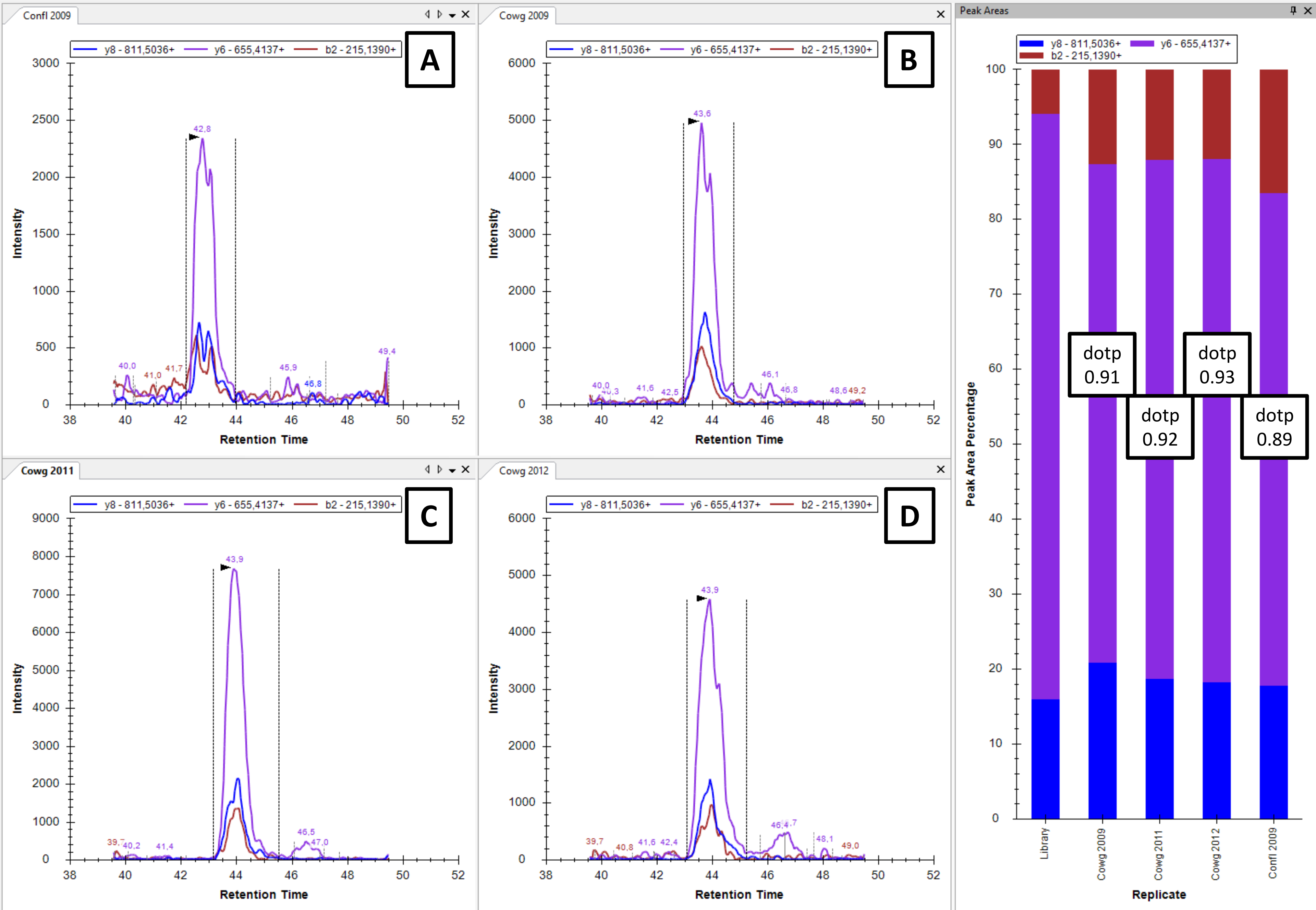

Peptide11-**AioA** -LIFTGIQTPTVR

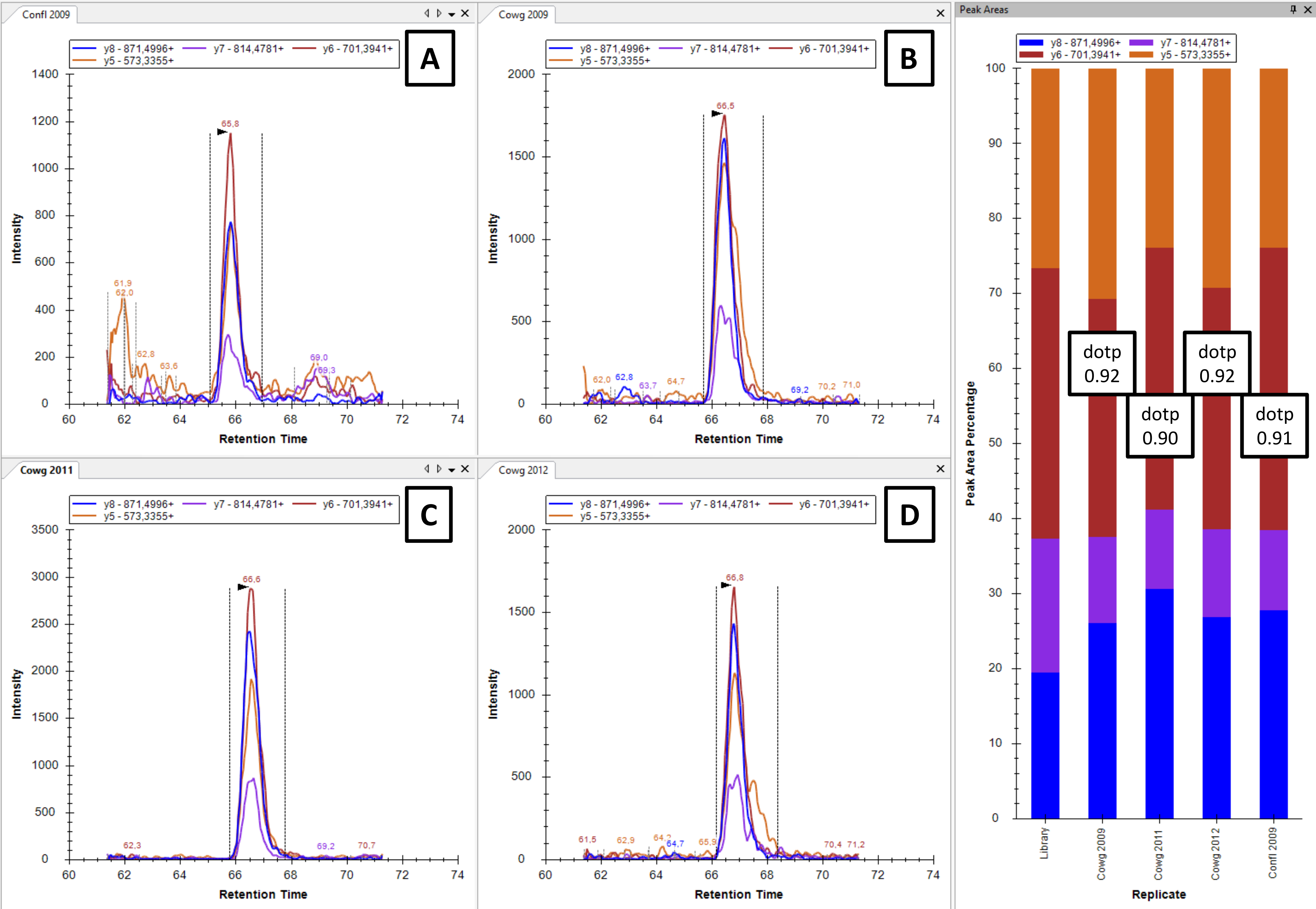

Peptide12-**AioB**-AVAVTGLIYGR

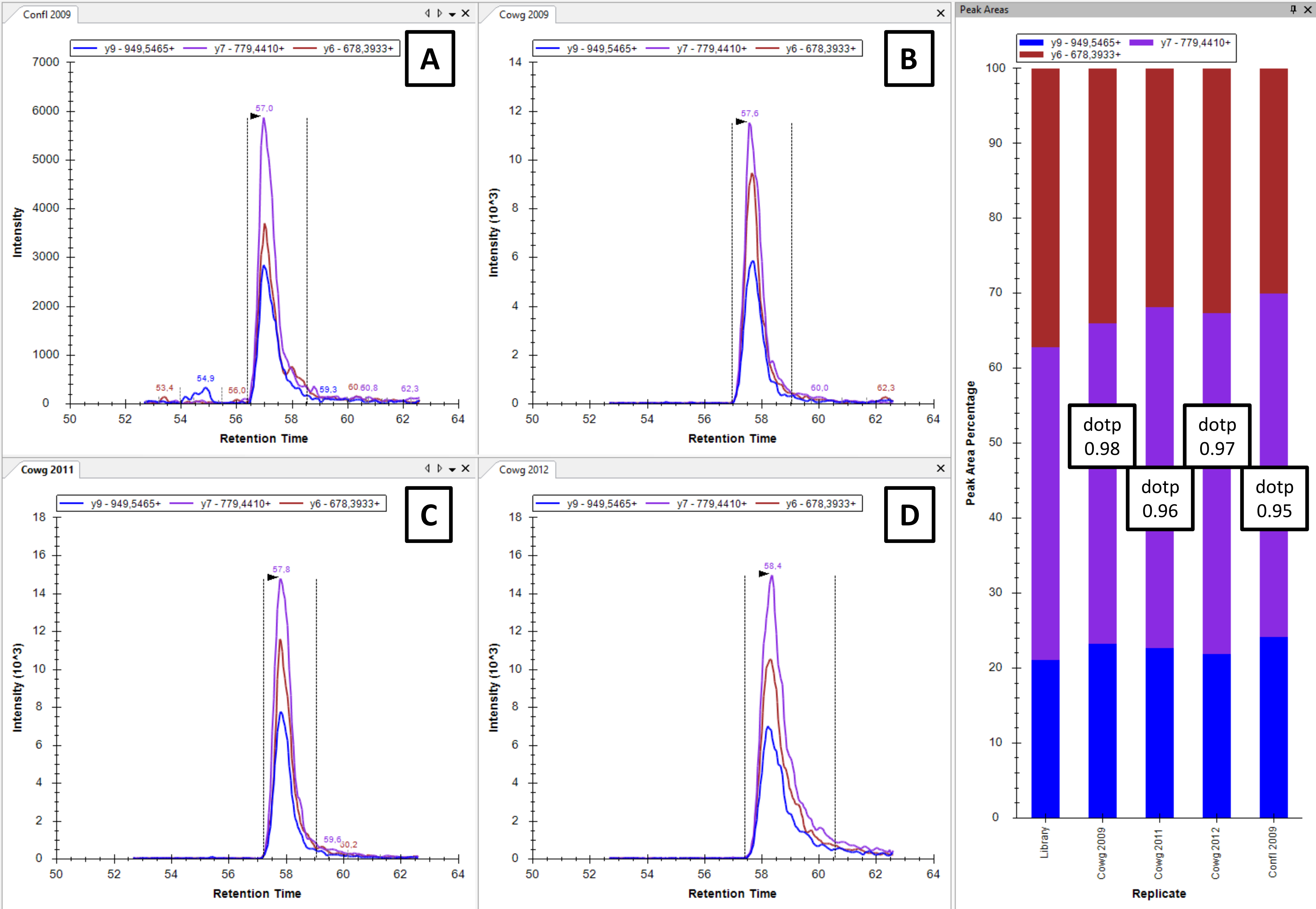

Peptide15-**RpoA**-SIQVEALGPLR

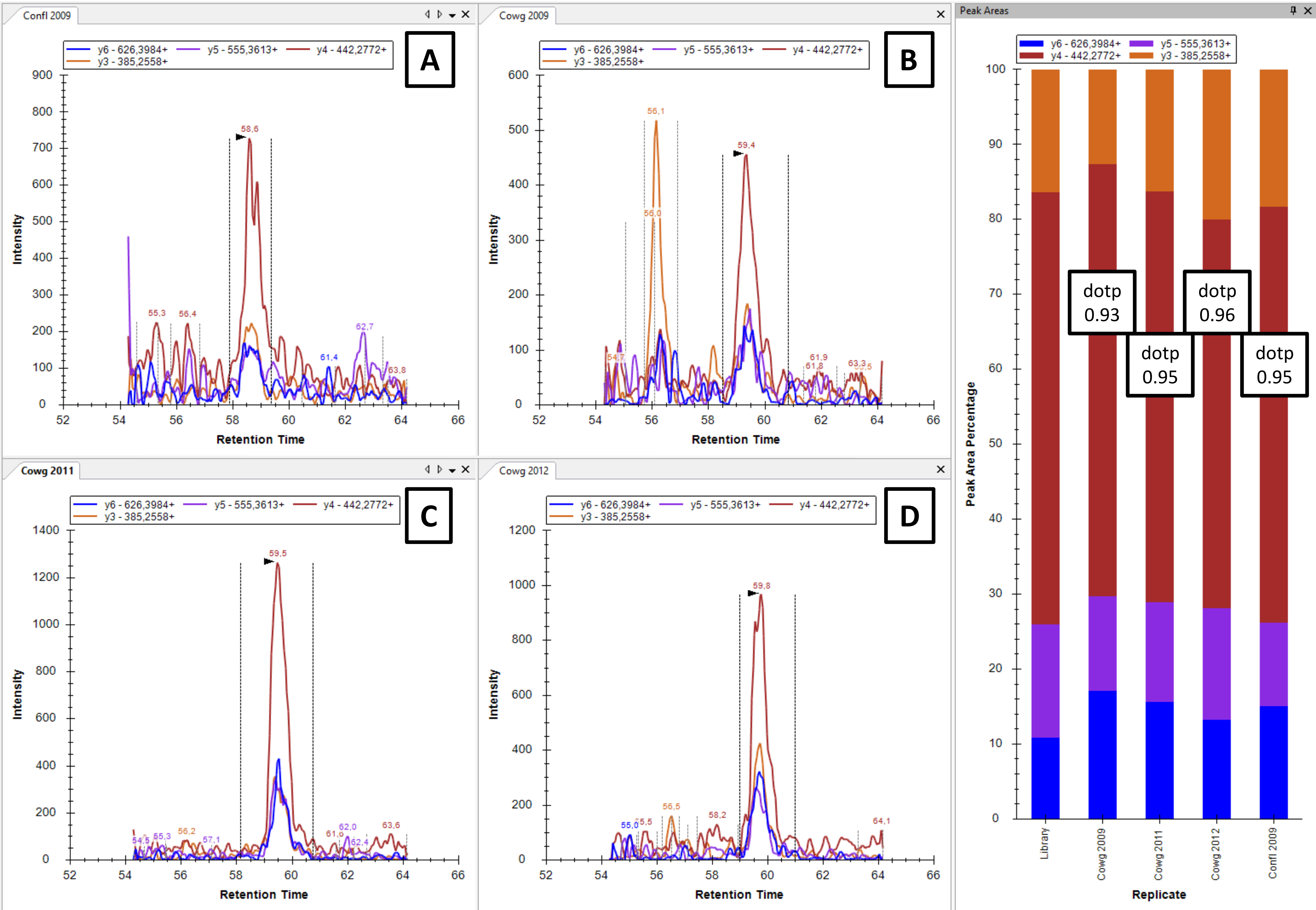

Supplement: Supplementary Figure 1 — Detection of the AioA/B and RpoA peptides using the SRM assay. Chromatograms were extracted with Skyline (MacLean et al., 2010). Two locations (A,B) were tested at three different time points (B–D). (A) CONF 2009; (B) COWG 2009; (C) COWG 2011; (D) COWG 2012. Each transition is indicated by one color. On the right, in the peak area windows, a dot p-value close to 1 indicates the existence of a very good correlation between the transitions and relative fragment intensities of the endogenous peptides and the reference library spectra. [file SupplementaryFigure1.PDF]
