# Supplementary material for: Spatio-Temporal Detection of the Thiomonas Population and the Thiomonas Arsenite Oxidase Involved in Natural Arsenite Attenuation Processes in the Carnoulès Acid Mine Drainage
Source: Front Cell Dev Biol. 2016 Feb 1;4:3. doi: 10.3389/fcell.2016.00003 (PMC4734075; doi:10.3389/fcell.2016.00003)
Supplement: Supplementary Table 3 — Estimated OTU richness, diversity indices and estimated sample coverage for each 16S rRNA libraries using pyrosequencing analysis. Results are presented for the normalized data, randomly resampled to have an equal sample size. [file Table3.DOCX]

**Supplementary Table 3.** Estimated OTU richness, diversity indices and estimated sample coverage for each 16S rRNA libraries using pyrosequencing analysis. Results are presented for the normalized data, randomly resampled to have an equal sample size.

| **Sampling**  **stations** | **No. of sequences**  **after quality filtering** | **No. of**  **Normalized sequences** | **No. of**  **OTUs^a^** | **Coverage^b^** | **Shannon^c^** | **Chao1** |
| --- | --- | --- | --- | --- | --- | --- |
| S1 Jun. 2011 n1 | 12260 | 2285 | 71 | 99 | 2.86 (2.81 ; 2.92) | 88 (76 ; 123) |
| S1 Jun. 2011 n2 | 2285 | 2285 | 41 | 99 | 1.91 (1.85 ; 1.96) | 50 (43 ; 77) |
| S1 Jan. 2012 n1 | 10122 | 2285 | 37 | 99 | 0.78 (0.72 ; 0.84) | 57 (43 ; 103) |
| S1 Jan. 2012 n2 | 13711 | 2285 | 40 | 99 | 0.81 (0.74 ; 0.87) | 67 (48 ; 129) |
| COWG Jun. 2011 n1 | 10024 | 2285 | 98 | 99 | 3.35 (3.30 ; 3.41) | 127 (109 ; 172) |
| COWG Jun. 2011 n2 | 13281 | 2285 | 87 | 99 | 3.42 (3.37 ; 3.47) | 97 (90 ; 120) |
| COWG Jan. 2012 n1 | 12631 | 2285 | 97 | 98 | 2.17 (2.09 ; 2.24) | 127 (110 ; 165) |
| COWG Jan. 2012 n2 | 11949 | 2285 | 65 | 99 | 1.79 (1.72 ; 1.86) | 76 (68 ; 99) |
| CONF Jun. 2011 n1 | 18409 | 2285 | 379 | 96 | 5.40 (5.36 ; 5.45) | 445 (418 ; 490) |
| CONF Jun. 2011 n2 | 9613 | 2285 | 335 | 97 | 5.32 (5.28 ; 5.36) | 365 (351 ; 394) |
| CONF Jan. 2012 n1 | 13998 | 2285 | 360 | 95 | 4.91 (4.85 ; 4.97) | 439 (410 ; 487) |
| CONF Jan. 2012 n2 | 14324 | 2285 | 297 | 96 | 4.69(4.63 ; 4.75) | 367 (338 ; 417) |

^a^ OTUs were defined at 97% cutoff

^b^ Coverage: sum of probabilities of observed classes calculated as (1 - (n/N)), where n is the number of singleton sequences and N is the total number of sequences

^c^ Takes into account the number and evenness of species

Values in brackets are 95% confidence intervals
