# Supplementary material for: Spatio-Temporal Detection of the Thiomonas Population and the Thiomonas Arsenite Oxidase Involved in Natural Arsenite Attenuation Processes in the Carnoulès Acid Mine Drainage
Source: Front Cell Dev Biol. 2016 Feb 1;4:3. doi: 10.3389/fcell.2016.00003 (PMC4734075; doi:10.3389/fcell.2016.00003)
Supplement: Supplementary Table 4 — Distribution of normalized sequences of bacterial 16S rRNA genes from each samples assigned to different phyla. [file Table4.DOCX]

**Supplementary Table 4.** Distribution of normalized sequences of bacterial 16S rRNA genes from each samples assigned to different phyla.

| **Phylum** | **Total** | | | | **S1** | | | | **COWG** | | | | **CONF** | | | |
| --- | --- | --- | --- | --- | --- | --- | --- | --- | --- | --- | --- | --- | --- | --- | --- | --- |
|  | **June 2011** | | **January 2012** | | **June 2011** | | **January 2012** | | **June 2011** | | **January 2012** | | **June 2011** | | **January 2012** | |
|  | **n1** | **n2** | **n1** | **n2** | **n1** | **n2** | **n1** | **n2** | **n1** | **n2** | **n1** | **n2** | **n1** | **n2** | **n1** | **n2** |
| ***Proteobacteria*** | **2875** | **2791** | **4949** | **4913** | 959 | 916 | 2039 | 2068 | 833 | 811 | 1423 | 1320 | 1083 | 1064 | 1487 | 1525 |
| **Other: unclassified** | **3144** | **3243** | **1490** | **1612** | 1215 | 1337 | 231 | 202 | 1305 | 1295 | 820 | 936 | 624 | 611 | 439 | 474 |
| ***Bacteroidetes*** | **226** | **224** | **126** | **134** | 0 | 0 | 0 | 0 | 0 | 3 | 1 | 1 | 226 | 221 | 125 | 133 |
| ***Acidobacteria*** | **170** | **223** | **108** | **76** | 19 | 14 | 5 | 4 | 24 | 39 | 8 | 10 | 127 | 170 | 95 | 62 |
| ***Actinobacteria*** | **185** | **140** | **103** | **63** | 36 | 5 | 3 | 1 | 43 | 50 | 4 | 2 | 106 | 85 | 96 | 60 |
| ***Firmicutes*** | **174** | **156** | **53** | **43** | 55 | 13 | 7 | 10 | 58 | 70 | 27 | 12 | 61 | 73 | 19 | 21 |
| ***Chlorobi*** | **45** | **38** | **3** | **3** | 0 | 0 | 0 | 0 | 11 | 6 | 2 | 3 | 34 | 32 | 1 | 0 |
| ***Nitrospirae*** | **2** | **11** | **17** | **10** | 0 | 0 | 0 | 0 | 2 | 11 | 0 | 1 | 0 | 0 | 17 | 9 |
| ***Spirochaetes*** | **15** | **11** | **0** | **0** | 0 | 0 | 0 | 0 | 0 | 0 | 0 | 0 | 15 | 11 | 0 | 0 |
| ***Gemmatimonadetes*** | **8** | **7** | **1** | **1** | 1 | 0 | 0 | 0 | 0 | 0 | 0 | 0 | 7 | 7 | 1 | 1 |
| **TM7** | **9** | **4** | **0** | **0** | 0 | 0 | 0 | 0 | 9 | 0 | 0 | 0 | 0 | 4 | 0 | 0 |
| ***Verrucomicrobia*** | **0** | **5** | **2** | **0** | 0 | 0 | 0 | 0 | 0 | 0 | 0 | 0 | 0 | 5 | 2 | 0 |
| ***Fusobacteria*** | **1** | **1** | **1** | **0** | 0 | 0 | 0 | 0 | 0 | 0 | 0 | 0 | 1 | 1 | 1 | 0 |
| ***Chlamydiae*** | **0** | **0** | **2** | **0** | 0 | 0 | 0 | 0 | 0 | 0 | 0 | 0 | 0 | 0 | 2 | 0 |
| ***Cyanobacteria*** | **1** | **1** | **0** | **0** | 0 | 0 | 0 | 0 | 0 | 0 | 0 | 0 | 1 | 1 | 0 | 0 |
