# Supplementary material for: Spatio-Temporal Detection of the Thiomonas Population and the Thiomonas Arsenite Oxidase Involved in Natural Arsenite Attenuation Processes in the Carnoulès Acid Mine Drainage
Source: Front Cell Dev Biol. 2016 Feb 1;4:3. doi: 10.3389/fcell.2016.00003 (PMC4734075; doi:10.3389/fcell.2016.00003)
Supplement: Supplementary Table 5 — Distribution of normalized sequences of bacterial 16S rRNA genes of the phylum of Proteobacteria assigned to different orders. [file Table5.DOCX]

**Supplementary Table 5.** Distribution of normalized sequences of bacterial 16S rRNA genes of the phylum of *Proteobacteria* assigned to different orders.

| **Order of *Proteobacteria*** | **Total** | | | | **S1** | | | | **COWG** | | | | **CONF** | | | |
| --- | --- | --- | --- | --- | --- | --- | --- | --- | --- | --- | --- | --- | --- | --- | --- | --- |
|  | **June 2011** | | **January 2012** | | **June 2011** | | **January 2012** | | **June 2011** | | **January 2012** | | **June 2011** | | **January 2012** | |
|  | **n1** | **n2** | **n1** | **n2** | **n1** | **n2** | **n1** | **n2** | **n1** | **n2** | **n1** | **n2** | **n1** | **n2** | **n1** | **n2** |
| ***Gallionellales*** | **866** | **760** | **3225** | **3162** | 553 | 689 | 1996 | 1994 | 310 | 68 | 1215 | 1147 | 3 | 3 | 14 | 21 |
| ***Unclassified total*** | **651** | **609** | **415** | **371** | 114 | 39 | 8 | 14 | 159 | 169 | 92 | 96 | 378 | 401 | 315 | 261 |
| ***Acidithiobacillales*** | **469** | **592** | **37** | **30** | 217 | 138 | 3 | 3 | 252 | 454 | 28 | 24 | 0 | 0 | 6 | 3 |
| ***Burkholderiales*** | **253** | **286** | **294** | **251** | 52 | 42 | 20 | 27 | 33 | 49 | 35 | 25 | 168 | 195 | 239 | 199 |
| ***Rhodospirillales*** | **130** | **118** | **255** | **270** | 9 | 3 | 0 | 4 | 33 | 25 | 23 | 12 | 88 | 90 | 232 | 254 |
| ***Xanthomonadales*** | **55** | **72** | **183** | **228** | 8 | 5 | 5 | 2 | 12 | 20 | 13 | 9 | 35 | 47 | 165 | 217 |
| ***Rhizobiales*** | **49** | **40** | **130** | **178** | 1 | 0 | 1 | 1 | 3 | 3 | 2 | 1 | 45 | 37 | 127 | 176 |
| ***Sphingomonadales*** | **24** | **26** | **105** | **152** | 0 | 0 | 0 | 1 | 0 | 0 | 2 | 0 | 24 | 26 | 103 | 151 |
| ***Legionellales*** | **60** | **56** | **98** | **85** | 0 | 0 | 6 | 22 | 27 | 22 | 7 | 6 | 33 | 34 | 85 | 57 |
| ***Desulfuromonadales*** | **102** | **102** | **9** | **10** | 0 | 0 | 0 | 0 | 1 | 0 | 0 | 0 | 101 | 102 | 9 | 10 |
| ***Neisseriales*** | **65** | **38** | **56** | **42** | 0 | 0 | 0 | 0 | 2 | 1 | 0 | 0 | 63 | 37 | 56 | 42 |
| ***Rhodobacterales*** | **48** | **23** | **40** | **42** | 0 | 0 | 0 | 0 | 1 | 0 | 2 | 0 | 47 | 23 | 38 | 42 |
| ***Pseudomonadales*** | **11** | **3** | **41** | **35** | 4 | 0 | 0 | 0 | 0 | 0 | 2 | 0 | 7 | 3 | 39 | 35 |
| ***Caulobacterales*** | **18** | **11** | **19** | **19** | 0 | 0 | 0 | 0 | 0 | 0 | 2 | 0 | 18 | 11 | 17 | 19 |
| ***Myxococcales*** | **23** | **27** | **7** | **2** | 0 | 0 | 0 | 0 | 0 | 0 | 0 | 0 | 23 | 27 | 7 | 2 |
| ***Methylophilales*** | **0** | **0** | **15** | **11** | 0 | 0 | 0 | 0 | 0 | 0 | 0 | 0 | 0 | 0 | 15 | 11 |
| ***Rhodocyclales*** | **9** | **3** | **5** | **4** | 0 | 0 | 0 | 0 | 0 | 0 | 0 | 0 | 9 | 3 | 5 | 4 |
| ***Campylobacterales*** | **3** | **0** | **6** | **11** | 0 | 0 | 0 | 0 | 0 | 0 | 0 | 0 | 3 | 0 | 6 | 11 |
| ***Aeromonadales*** | **7** | **5** | **2** | **3** | 0 | 0 | 0 | 0 | 0 | 0 | 0 | 0 | 7 | 5 | 2 | 3 |
| ***Rickettsiales*** | **5** | **10** | **0** | **0** | 0 | 0 | 0 | 0 | 0 | 0 | 0 | 0 | 5 | 10 | 0 | 0 |
| ***Desulfobacterales*** | **5** | **8** | **0** | **0** | 0 | 0 | 0 | 0 | 0 | 0 | 0 | 0 | 5 | 8 | 0 | 0 |
| ***Syntrophobacterales*** | **10** | **0** | **0** | **0** | 0 | 0 | 0 | 0 | 0 | 0 | 0 | 0 | 10 | 0 | 0 | 0 |
| ***Enterobacteriales*** | **1** | **0** | **2** | **5** | 0 | 0 | 0 | 0 | 0 | 0 | 0 | 0 | 1 | 0 | 2 | 5 |
| ***Desulfovibrionales*** | **6** | **0** | **1** | **0** | 0 | 0 | 0 | 0 | 0 | 0 | 0 | 0 | 6 | 0 | 1 | 0 |
| ***Bdellovibrionales*** | **2** | **1** | **0** | **1** | 0 | 0 | 0 | 0 | 0 | 0 | 0 | 0 | 2 | 1 | 0 | 1 |
| ***Pasteurellales*** | **3** | **1** | **0** | **0** | 1 | 0 | 0 | 0 | 0 | 0 | 0 | 0 | 2 | 1 | 0 | 0 |
| ***Methylococcales*** | **0** | **0** | **2** | **0** | 0 | 0 | 0 | 0 | 0 | 0 | 0 | 0 | 0 | 0 | 2 | 0 |
| ***Thiotrichales*** | **0** | **0** | **2** | **0** | 0 | 0 | 0 | 0 | 0 | 0 | 0 | 0 | 0 | 0 | 2 | 0 |
| ***Alteromonadales*** | **0** | **0** | **0** | **1** | 0 | 0 | 0 | 0 | 0 | 0 | 0 | 0 | 0 | 0 | 0 | 1 |
