# Supplementary material for: Spatio-Temporal Detection of the Thiomonas Population and the Thiomonas Arsenite Oxidase Involved in Natural Arsenite Attenuation Processes in the Carnoulès Acid Mine Drainage
Source: Front Cell Dev Biol. 2016 Feb 1;4:3. doi: 10.3389/fcell.2016.00003 (PMC4734075; doi:10.3389/fcell.2016.00003)
Supplement: Supplementary file 8 [file Supplementarydata.DOCX]

**Supplementary Material and Methods**

**Liquid Chromatography (LC) Selected Reaction Monitoring (SRM) assay.** An MS/MS spectral library was acquired on the mixture of the sixteen heavy labelled synthetic peptides injected for nanoLC-MS/MS analysis on an Agilent 1100 series HPLC-Chip/MS system (Agilent Technologies, Palo Alto, USA) coupled to an HCT Plus ion trap (Bruker Daltonics, Bremen, Germany). The column used was a chip C18 SB-ZORBAX, 300 Å (75 µm x 150 mm, 5 µm).The solvent system consisted of 2% acetonitrile, 0.1% formic acid in water (solvent A) and 2% water, 0.1% formic acid in acetonitrile (solvent B). Elution was performed at a flow rate of 300 nL/min with a 8-40% gradient (solvent B) over 30 first minutes followed by a 70% stage (solvent B) over 3 min before the reconditioning of the column at 92% of solvent A. The voltage applied to the capillary cap was optimized to -1,750 V. The system was operated with automatic switching between MS and MS/MS modes. The three most abundant peptides, preferring doubly charged ions, were selected on each MS spectrum for further isolation and fragmentation. The MS/MS scanning was performed in the ultrascan resolution mode at a scan rate of 26.000m/z per second. A total of 6 scans were averaged to obtain an MS/MS spectrum. The complete system was fully controlled by ChemStation (Agilent Technologies) and EsquireControl (Bruker Daltonics) software.

Mass data collected during nano-LC-MS/MS analysis were processed, converted into .mgf files, and interpreted using a local Mascot server (version 2.4.1, Matrix Science, London, U.K.). Searches were performed against a database containing all peptides of interest assembled as a chimeric fake protein. Searches were performed with a tolerance on mass measurements of 0.25 Da in both MS and MS/MS modes. In Skyline, precursor and product ion masses were selected in the spectral library with the following settings : monoisotopic masses, precursor charges +2 and +3, product ion charges +1, +2, from m/z > precursor and up to 6 y- or b- ions, N-terminal fragments to proline were allowed. In order to keep only the most intense transitions and minimize interferences, this large list of transitions was refined using a pool of bacterial strains proteins spiked with the heavy peptides mixture analyzed by microLC-SRM in exactly the same conditions as the samples. These analyzes also allowed to determine peptides retention time. At the end, for each peptide, at least three transitions were monitored in order to identify the peptide and the quantification was done only on non-interfered transitions. The collision energies were also experimentally and individually optimized using the heavy standard peptides mixture injected in microLC-SRM by testing nine values centred on the calculated value suggested by the supplier. The complete list of measured transitions with their retention times and optimized collision energies is provided in Supplementary Table 1.

**Sample preparation and protein digestion:**

Reduction was made with 1.75 µL of 700 mM dithiothreitol (DTT) for 30 min at 37°C, followed by alkylation with 6 µL of 700 mM iodoacetamide (IAA) for 1 h at room temperature, in the dark. In order to quench residual IAA, new 1.75 µL of DTT were added. Then samples were diluted to 1 M urea with 750 µL of 0.1 M fresh NH_4_HCO_3_. Digestion was performed overnight at 37°C with 0.5 µg of trypsin, and stopped with 5 µL HCOOH. All quantities were multiplied by two in order to constitute the QC sample. Sediments samples contained only a maximum of 15 µg of proteins in 300 µL. Their volume was first reduced to 100 µL under speed vacuum. Proteins were then solubilized in 200 µL of 8 M urea and 0.1 M NH_4_HCO_3_ and spiked with the heavy labeled standard peptides mixture. Reduction was performed with 3.5 µL of 700 mM dithiothreitol (DTT) for 30 min at 37°C, followed by alkylation with 12 µL of 700 mM iodoacetamide (IAA) for 1 h at room temperature, in the dark. In order to quench residual IAA, new 3.5 µL of DTT were added. Then samples were diluted to 1 M urea with 1500 µL of fresh 0.1 M NH_4_HCO_3_. Digestion was made overnight at 37°C with 0.2 µg of trypsin, and stopped with 10 µL HCOOH. All digested samples were desalted on Sep-Pak C18 50 mg cartridges (Waters, Milford, MA, USA). Cartridges were washed with 2 mL of methanol, 2 mL of acetonitrile (ACN) and equilibrated with 3 mL of 0.1% HCOOH. Samples were loaded, washed with 3 mL of 0.1% HCOOH and eluted with 600 µL of 60% ACN, 0.1% HCOOH (v/v). Bacterial strains samples’ volume was reduced to 50 µL with speed vacuum. Sediments samples’ volume was reduced to less than 7 µL in order to inject the entire sample in one time.
